# ﻿Systematic review of the genus *Pseudognaptorina* Kaszab, 1977 (Coleoptera, Tenebrionidae, Blaptinae, Blaptini) from the Qinghai-Xizang Plateau, with description of six new species

**DOI:** 10.3897/zookeys.1234.137739

**Published:** 2025-04-08

**Authors:** Xiu-Min Li, Bao-Yue Zhang, Ji-Gang Li, Zhao Pan

**Affiliations:** 1 Key Laboratory of Zoological Systematics and Application of Hebei Province, College of Life Sciences, Institute of Life Science and Green Development, Hebei University, Baoding 071002, China Hebei University Baoding China

**Keywords:** China, COI gene, darkling beetle, morphology

## Abstract

The genus *Pseudognaptorina*, with four described species, is endemic to the Qinghai-Xizang Plateau. In this study, *Pseudognaptorina* is reviewed based on a combination of molecular and morphological datasets. A preliminary phylogenetic tree is reconstructed based on COI sequences of four related genera within the subtribe Gnaptorinina. Additionally, the geographical distribution of *Pseudognaptorina* is presented. Six new species are described and illustrated: *P.banbarica* X.-M. Li, **sp. nov.**, *P.himalayana* X.-M. Li, **sp. nov.**, *P.migana* X.-M. Li, **sp. nov.**, *P.oblonga* X.-M. Li, **sp. nov.**, *P.rectangularis* X.-M. Li, **sp. nov.**, and *P.reni* X.-M. Li, **sp. nov.** This work provides valuable molecular, morphological, and distributional data for the study of species evolution in the subtribe Gnaptorinina.

## ﻿Introduction

The subtribe Gnaptorinina Medvedev, 2001 belongs to the tribe Blaptini Leach, 1815 within the subfamily Blaptinae. It consists of 189 species in 12 genera, which are mainly distributed at high-elevations of the Qinghai-Xizang Plateau. These genera are *Agnaptoria* Reitter, 1887 (36 species and subspecies), *Asidoblaps* Fairmaire, 1886 (56 species), *Blaptogonia* Medvedev, 1998 (five species), *Colasia* Koch, 1965 (seven species and subspecies), *Gnaptorina* Reitter, 1887 (39 species), *Itagonia* Reitter, 1887 (24 species), *Montagona* Medvedev, 1998 (three species), *Nepalindia* Medvedev, 1998 (five species), *Pseudognaptorina* Kaszab, 1977 (four species), *Sintagona* Medvedev, 1998 (one species), *Tagonoides* Fairmaire, 1886 (eight species), and *Viettagona* Medvedev & Merkl, 2003 (one species) (Medvedev and Merkl 2002; Medvedev 2004; Ren et al. 2016; Li et al. 2018, 2019; Chigray 2019; Bai et al. 2020, 2023; Nabozhenko and Chigray 2020; Ji et al. 2024).

**Figure 1.**
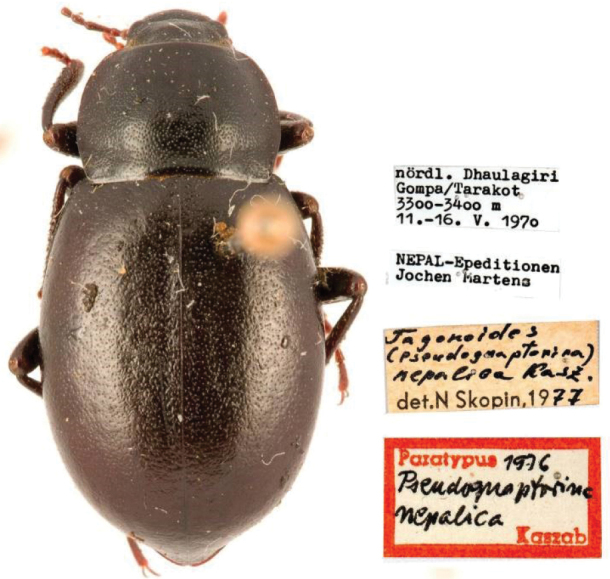
*Pseudognaptorinanepalica* Kaszab, 1977, male.

*Pseudognaptorina* was established by Kaszab (1977), with the type species *P.nepalica* Kaszab, 1977 (Fig. 1) from Nepal. Later, three species (*P.exsertogena* Shi, Ren & Merkl, 2005, *P.flata* Liu & Ren, 2009, and *P.obtusa* Shi, Ren & Merkl, 2005) were described from Xizang and Sichuan, China (Shi et al. 2005; Liu and Ren 2009). *Pseudognaptorina* is morphologically similar to the genus *Gnaptorina* but differs in the following characters: protibial spurs subequal in length; ventral surface of male protarsomeres I–III and at least mesotarsomere I with hair brushes; aedeagus with moderately elongate parameres, at least twice as long as wide. *Pseudognaptorina* is also related to *Montagona* but can be distinguished by the following characters: epipleural carina visible in dorsal view at apex; elytra without longitudinal carina or smooth bulge; ventral surface of male protarsomeres I–III and at least mesotarsomere I with hair brushes; lobes of ovipositor transverse. Medvedev (2009) proposed that the genus *Pseudognaptorina* likely comprises only the type species, *P.nepalica*. The generic taxonomic status of *P.exsertogena* and *P.obtusa* is doubtful, as these two species are found in east-central Xizang, China, which is considerably distant from the type locality of the type species. Notably, *P.flata* has been discovered in Sichuan, China, which is geographically close to *P.exsertogena* (Liu and Ren 2009). To date, the genus *Pseudognaptorina* has not been studied using molecular data. Consequently, the taxonomic status of these species requires evaluation within the framework of molecular phylogeny.

In this study, six new species are described and illustrated. In addition, we construct a phylogenetic tree for four related genera to investigate the monophyly of the genus *Pseudognaptorina*. This study provides valuable molecular and distributional data on *Pseudognaptorina*, which can be used to study the richness of endemic insects in the surrounding areas of the Qinghai-Xizang Plateau.

## ﻿Materials and methods

### ﻿Morphological examination

Nine species (108 specimens) of the genus *Pseudognaptorina* were examined for this study and deposited at the Museum of Hebei University, Baoding, China (**MHBU**) and Hungarian Natural History Museum, Budapest, Hungary (**HNHM**). The specimens were photographed using a Canon EOS 5D Mark III (Canon Inc., Tokyo, Japan) with a Laowa FF 100 mm F2.8 CA-Dreamer Macro 2 × lens, or Laowa FF 25 mm F2.8 Ultra Macro 2.5–5 × lens (Anhui Changgeng Optics Technology Co., Ltd, Hefei, China).

Label data are presented verbatim. A slash (/) separates text on lines of the label.

### ﻿Taxon sampling, DNA extraction, PCR amplification, and sequencing

Specimens were collected in the field on the Qinghai-Xizang Plateau. Molecular data were collected from 148 individuals and includes previously published sequences from 72 individuals of the genus *Gnaptorina* (Li et al. 2021; Ji et al. 2024).

DNA was extracted from the leg muscle tissue of adults using the Insect DNA isolation Kit (BIOMIGA, Dalian, China) following the manufacturer’s protocols. The DNA extracted was stored at -20 °C. The fragment of the mitochondrial molecular marker (cytochrome oxidase subunit I, COI) was amplified with the primers F 2183 and R 3014 (Folmer et al. 1994). The profile of the PCR amplification consisted of an initial denaturation step at 94 °C for 4 min, 35 cycles of denaturation at 94 °C for 1 min, annealing for 45 s, an extension at 72 °C for 1 min, and a final 8 min extension step at 72 °C. PCR was performed using TaKaRa Ex Taq (TaKaRa, Dalian, China). PCR products were subsequently checked by 1% agarose gel electrophoresis and sequenced at General Biol Co. (Chuzhou, China).

### ﻿Phylogenetic analyses

In total, 148 sequences from 70 species were used for the phylogenetic analyses including 72 new sequences from four genera: *Agnaptoria*, *Asidoblaps*, *Gnaptoriana*, and *Pseudognaptorina*. Detailed information on the new samples in this study is provided in Suppl. material 1, and the previously published sequence numbers were labeled on the phylogenetic tree. The tribe Platyscelidini was selected as the outgroup, which was considered to be most closely related to the tribe Blaptini (Kamiński et al. 2021).

Phylogenetic analysis was based on the COI gene fragment using maximum likelihood (ML). A best-fit model was tested according to the corrected Akaike’s Information Criterion (AICc) using ModelFinder (included in IQ-TREE) with PhyloSuite v. 1.2.2 (Zhang et al. 2020). The ML tree search was performed in IQ-TREE v. 1.6.8 (Nguyen et al. 2015). The ML tree was inferred using an edge-linked partition model for 5000 ultrafast bootstraps (1000 replicates) (Minh et al. 2013). Support for each node is represented by ultrafast bootstrap values (uBV).

## ﻿Results

### ﻿Morphological study

Key to males of *Pseudognaptorina* species

**Table:** 

1	Lateral margins of pronotum regularly arcuate. Elytral surface densely covered with rather smooth punctation, without coarse wrinkles. Ventral surface of protarsomeres I–II with hair brushes, protarsomere III with small hairy tuft; mesotarsomere I with hair brushes, mesotarsomere II with small hairy tuft	***P.nepalica***
–	Lateral margins of pronotum arcuate at anterior half. Elytra densely covered with indistinct punctation and coarse wrinkles. Ventral surface of protarsomeres I–III with hair brushes, mesotarsomeres I–III with hair brushes or tufts	**2**
2	Pronotum transverse, surface flatted. Ventral surface of mesotarsomeres I–II with hair brushes, mesotarsomere III with small hairy tuft	***P.rectangularis* sp. nov.**
–	Pronotum transverse, surface slightly convex. Ventral surface of mesotarsomeres I–III with hair brushes	**3**
3	Posterior angles of pronotum slightly obtuse. Parameres strongly elongate, 2.78 times as long as wide	***P.obtusa***
–	Posterior angles of pronotum almost rectangular. Parameres not strongly elongate	**4**
4	Lateral margins of pronotum arcuately narrowed at basal 2/3	***P.flata***
–	Lateral margins of pronotum arcuately narrowed at basal 1/2	**5**
5	Surface of elytra with fine punctures and irregular wrinkles	***P.oblonga* sp. nov.**
–	Surface of elytra with fine punctures and without regular wrinkles	**6**
6	Antennomeres VIII–X nearly cylindrical	**7**
–	Antennomeres VIII–X nearly spherical	**8**
7	Ventral surface of mesotarsomeres I–II with hair brushes, mesotarsomere III with small, hairy tuft	***P.banbarica* sp. nov.**
–	Ventral surface of mesotarsomeres I–III with hair brushes	**9**
8	Pronotum 1.28 times as wide as long. Surface of elytra with fine punctures and irregular wrinkles	***P.exsertogena***
–	Pronotm 1.50 times as wide as long. Surface of elytra with fine punctures and without regular wrinkles	***P.reni* sp. nov.**
9	Pronotum 1.34 times as wide as long, lateral margins arcuately narrowed at basal 2/3. Ventral surface of mesotarsomeres I–III with hair brushes	***P.migana* sp. nov.**
–	Pronotum 1.29 times as wide as long, lateral margins of pronotum regularly arcuate. Mesotarsomeres I–II with hair brushes, mesotarsomere III with small, hairy tuft	***P.himalayana* sp. nov.**

#### ﻿Key to females of *Pseudognaptorina* species

**Table:** 

1	Antennae long, reaching base of pronotum posteriad	**2**
–	Antennae short, not reaching base of pronotum posteriad	**3**
2	Pronotum 1.38 times as wide as long. Ratio of width at anterior margin to its maximum width and to width at posterior margin 0.58: 1.00: 0.89	***P.nepalica***
–	Pronotum 1.56 times as wide as long. Ratio of width at anterior margin to its maximum width and width at posterior margin 0.55: 1.00: 0.97	***P.reni* sp. nov.**
3	Protibial spurs rounded apically	***P.banbarica* sp. nov.**
–	Protibial spurs small and pointed	**4**
4	Posterior angles of pronotum slightly obtuse	**5**
–	Posterior angles of pronotum almost rectangular	**6**
5	Elytra wider (1.36 times as long as wide), densely covered with irregular wrinkles	***P.flata***
–	Elytra elongate-oval (1.28–1.29 times as long as wide), sparsely covered with fine punctures and irregular wrinkles	***P.obtusa***
6	Antennomere VII short. Pronotum slightly convex	***P.exsertogena***
–	Antennomere VII long. Pronotum flattened	**7**
7	Pronotum with fine punctation; stem of spiculum long	***P.migana* sp. nov.**
–	Pronotum with very dense punctation; stem of spiculum short	***P.oblonga* sp. nov.**

##### ﻿Genus Pseudognaptorina

Taxon classificationAnimaliaColeopteraTenebrionidae

Kaszab, 1977

E8192B04-C2A0-5158-BCC8-9965DCE3D194

Pseudognaptorina Kaszab, 1977: 250; Shi et al. 2005: 163; Liu and Ren 2009.

###### Type species.

*Pseudognaptorinanepalica* Kaszab, 1977, by original designation, by monotypy.

###### Generic diagnosis.

Antennomere VII narrower than VIII; epipleural carina visible in dorsal view at basal part and apex; all tibiae narrow, dilated apically, tarsi slender, ventral surface of male protarsomeres I–III and at least mesotarsomeres I–II with hair brushes; protibial spurs subequal in length; parameres moderately elongate, at least 1.8 times as long as wide; apical part of ovipositor short, 1–1.4 times as long as wide, lobes transverse.

###### Distribution.

Nepal and China.

##### ﻿ Pseudognaptorina exsertogena

Taxon classificationAnimaliaColeopteraTenebrionidae

Shi, Ren & Merkl, 2005

A8DFE6E4-244E-5B31-9310-1A4C23664A95

###### Type material examined.

***Holotype***: China • ♂ (MHBU XZ04062848): Yangbajain, Damxung County, Xizang/ 30°06'N, 90°30'E/ 3700–4100 m/ 2004-VI-28/ Yi-Bin Ba & Ai-Min Shi leg. ***Paratypes***: China • 15♂♂, 23♀♀ (MHBU XZ04062843–04062847, 04062849–04062871): same as holotype; China • 4♂♂, 1♀ (MHBU XZ02070511–02070515): Maizhokunggar County, Xizang/ 29°48'N, 91°48'E/ 4100 m/ 2002-VII-5/ Guo-Dong Ren leg.

###### Distribution.

Xizang, China.

##### ﻿ Pseudognaptorina flata

Taxon classificationAnimaliaColeopteraTenebrionidae

Liu & Ren, 2009

0675700D-8976-56B6-ADE4-7BC63F8065F4

###### Type material examined.

***Holotype***: China • ♂ (MHBU XZ08071698): Batang, Sichuan/ 30°07' N, 99°02' E/ 3850 m/ 2008-VII-16/ Guo-Dong Ren leg. ***Paratype***: China • 1♀ (MHBU XZ08071697): same data as the holotype.

###### Distribution.

Sichuan, China.

##### ﻿ Pseudognaptorina nepalica

Taxon classificationAnimaliaColeopteraTenebrionidae

Kaszab, 1977

AC264C89-5AA8-5A4A-A470-CA292460D167

###### Type material examined.

***Paratypes***: 1♀ (HNHM SMF C 14534): “Nepal. Expeditionen Jochen Martens,” “Gompabei Tarakot/ 3300–3400 m/ 1970-V-11–16”, “Tagonoides (Pseudognaptorina) nepalica Kasz., det. N. Skopin, 1976”; 1♂ (HNHM SMF C 14539), “Dolpo, Tal der oberen Barbung Khola, zwischen Terang und Tukot/ 4000 m/ 1970-VI-19”; 1♂ (HNHM SMF C 14537), “Dolpo, Weg von Kangar nach Shimen/ 4300–4500 m/ 1973-VI-18”; 1♂ (HNHM SMF C 14532), “Dolpo, Tal der oberen Barbung Khola, Charka/ 4300–4400 m/ 20–25.VI.1973”.

###### Distribution.

Nepal.

##### ﻿ Pseudognaptorina obtusa

Taxon classificationAnimaliaColeopteraTenebrionidae

Shi, Ren & Merkl, 2005

B87728E7-F804-5F43-8EBC-BD2E62D3547E

###### Type material examined.

***Holotype***: China • ♂ (MHBU XZ04061237): Markam County, Xizang/ 29°36'N, 98°24'E/ 3800–4000 m/ 2004-VI-12/ Ai-Min Shi & Yi-Bin Ba leg. ***Paratypes***: China • 2♀♀ (MHBU XZ04061238–04061239): same data as holotype.

###### Distribution.

Xizang, China.

##### ﻿ Pseudognaptorina banbarica

Taxon classificationAnimaliaColeopteraTenebrionidae

X.-M. Li sp. nov.

75D5ED86-58C5-5B8D-96AD-C383659F6D82

https://zoobank.org/8BEDBC76-29B9-4860-93AA-B9B858BF2D73

###### Type materials.

***Holotype***: China • ♂ (MHBU HBU(E)339867): Marxog Township, Banbar County, Xizang/ 31°01.410' N, 94°37.152' E/ Alt. 4400 m/ 2019-VII-31/ Guo-Dong Ren, Ya-Lin Li & Xing-Long Bai leg. ***Paratypes***: China • 3♂♂, 7♀♀ (MHBU HBU(E)339868–339877): same data as holotype.

###### Description.

**Figure 2.**
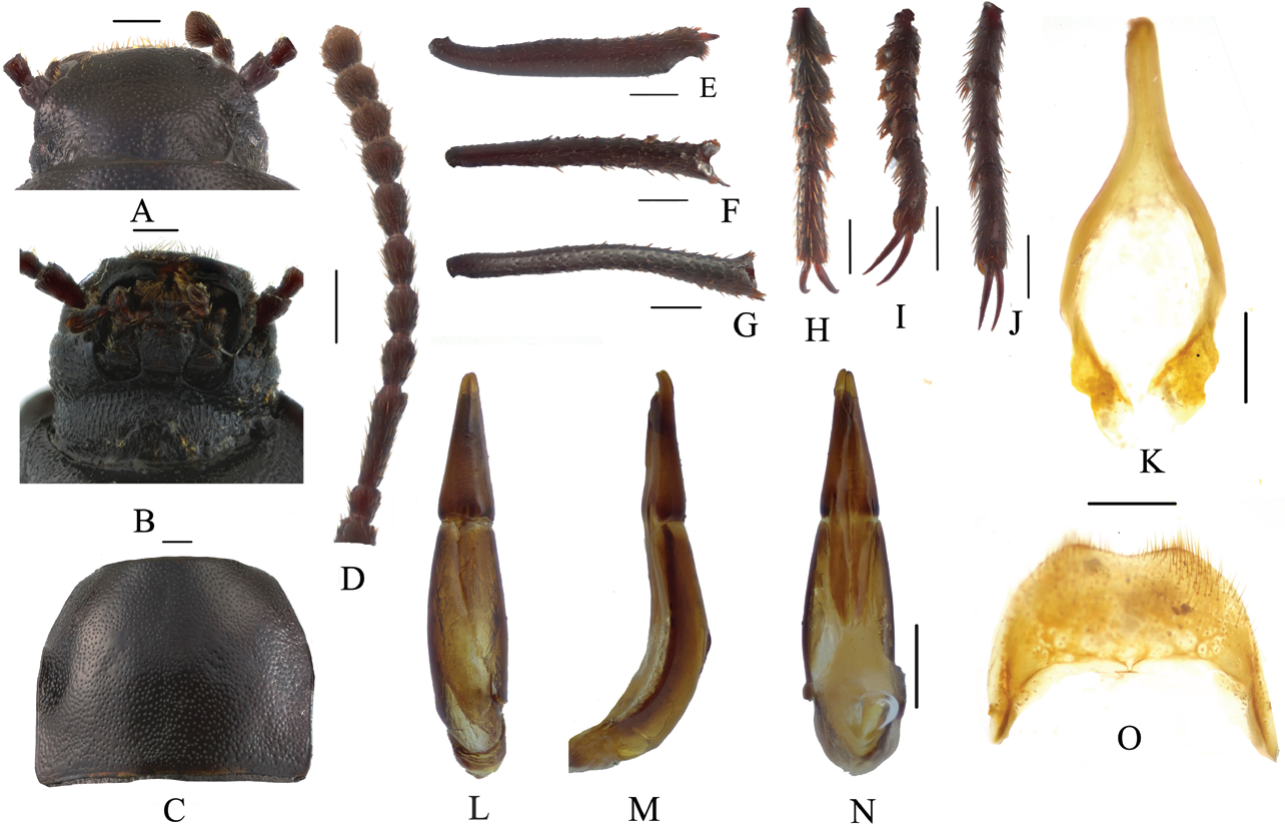
*Pseudognaptorinabanbarica* X.-M. Li, sp. nov., male **A** head, dorsal view **B** head, ventral view **C** pronotum **D** antenna **E** protibia **F** mesotibia **G** metatibia **H** protarsus **I** mesotarsus **J** metatarsus **K** spiculum gastrale **L–N** aedeagus **L** dorsal view **M** lateral view **N** ventral view **O** abdominal sternite VIII. Scale bars: 0.5 mm.

**Figure 3.**
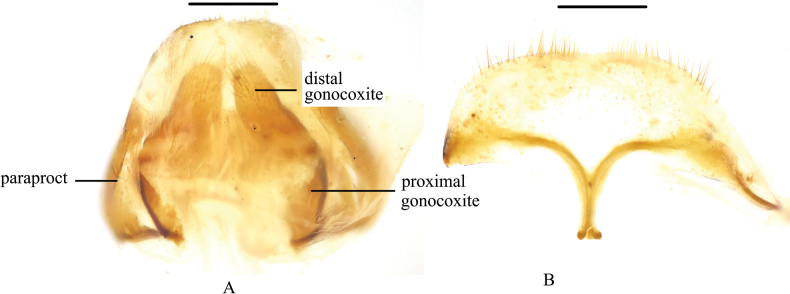
*Pseudognaptorinabanbarica* X.-M. Li, sp. nov., female **A** ovipositor **B** spiculum ventrale. Scale bars: 0.5 mm.

**Male** (Figs 2, 3A–C). Body length 10.5–11.0 mm, width 4.9–5.0 mm; shiny, black or brownish; antennae, palpi, and tarsi brown.

**Figure 4.**
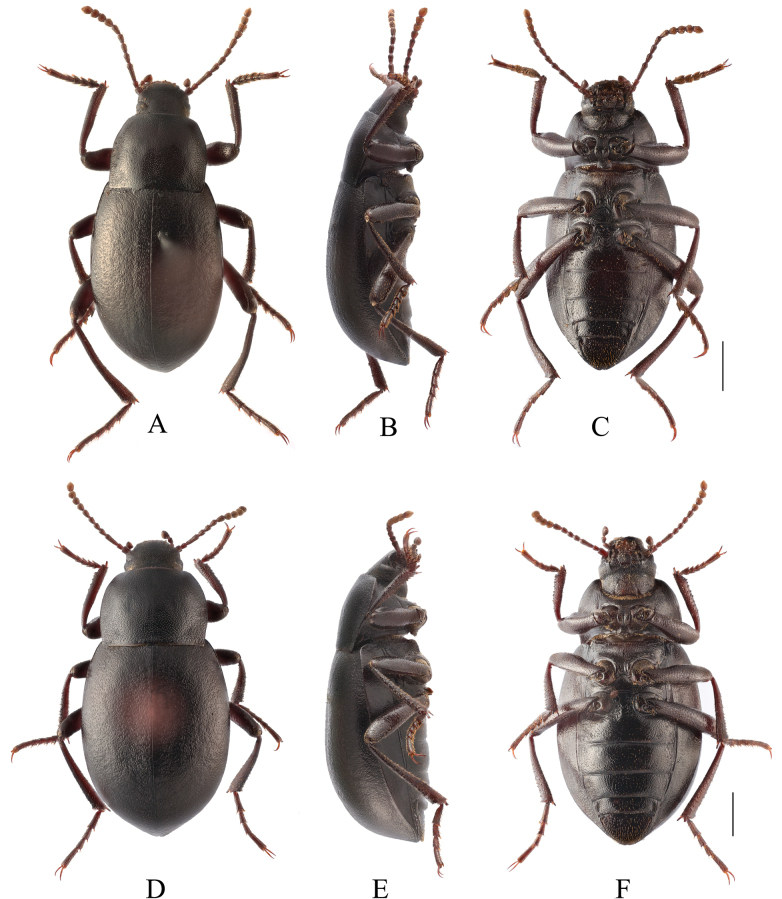
Habitus of *Pseudognaptorinabanbarica* X.-M. Li, sp. nov. **A–C** male, holotype **D–F** female, paratype **A, D** dorsal views **B, E** lateral views **C, F** ventral views. Scale bars: 2.0 mm.

***Head*** (Fig. 2A, B). Anterior margin of clypeus slightly sinuate. Head widest at eye level. Lateral margin of head with pair of projections between antennal base and oculus, brownish red. Genal margin arcuately converging before eyes. Eyes barely protruding beyond contour of head. Vertex flat or slightly convex, with uniform punctures. Antennae (Fig. 2D) slender and long, reaching beyond pronotal base when posteriorly extended, antennomeres III very long, 3.2 times as long as antennomeres II, antennomeres VIII–X oval, XI spindle-shaped. Length (width) ratio of antennomeres II–XI as follows: 10.9(10.0): 27.6(10.0): 13.9(10.0): 14.3(10.0): 15.0(10.0): 15.7(10.0): 12.6(12.7): 11.3(12.6): 11.7(14.3): 18.0(14.4).

***Prothorax*.** Pronotum (Fig. 2C) transverse, 1.28 times as wide as long, widest in middle, 1.78 times as wide as head. Ratio of width at anterior margin to its maximum width and posterior margin 0.60: 1.00: 0.97. Lateral margins of pronotum arcuately narrowing to anterior margin, bordered along entire length; posterior margin straight; anterior margin slightly emarginate; anterior angles widely obtuse-angled, posterior angles almost rectangular. Surface of pronotum slightly convex between lateral margins, covered with fine dense punctation. Hypomera covered shallow longitudinal wrinkles and granules. Prosternum before procoxae gently sloping. Prosternal process gently sloping behind procoxae, forming obtuse projection.

***Pterothorax*.** Elytra oblong-oval and convex, 1.37–1.39 times as long as wide, 1.38–1.39 times as wide as pronotum, widest in apical third. Dorsal surface of elytra passing into outer (deflexed) surface without traces of humeral carina. Outer margin of epipleura visible in dorsal view at basal third and apex. Surface of elytra with dense, rather smooth punctation and wrinkles almost vanishing on apical declivity.

***Legs*** (Fig. 2E–J). Femora and tibiae moderately thickened. Ratio of length (width) of pro-, meso-, and metatibiae: 43.8(6.6): 43.2(7.1): 69.6(8.0). Protibiae straight with shorter spurs, inner surface of protibiae slightly widened at basal third; mesotibiae slightly curved; metatibiae curved, narrow. Ventral surface of protarsomeres I–III with hairy brush; ventral surface of mesotarsomeres I–II with hairy brush. Ratio of length (width) of metatarsomeres I–IV: 24.0(9.8): 18.7(8.8): 15.6(8.3): 30.8(8.3).

***Abdomen*.** Abdominal ventrites rather sparsely covered with minute, pale, recumbent setae.

***Aedeagus*.** (Fig. 2K–O) Length of aedeagus 2.31 mm, width 0.42 mm; length of parameres 0.87 mm, width 0.29 mm. Slightly curved to ventral side apically in lateral view. Parameres strongly elongate, widest at base, regularly narrowing towards apex; outer margins slightly curved to ventral side apically in lateral view. Spiculum gastrale as in Fig. 2K. Posterior margin of abdominal sternite VIII sinuate (Fig. 2O).

**Female** (Figs 3A, B, 4D–F). Body larger and wider than male, length 12.6–14.4 mm, width 6.9–7.4 mm. Antennae shorter than male, not posteriorly reaching base of pronotum when posteriorly extended. Pronotum 1.52 times as wide as long, widest in middle, lateral margins subparallel from base to middle and arcuately narrowing toward anterior angles, sides of pronotum slightly convex, 1.79 times as wide as head, with very dense punctation. Elytra oval, more convex than male, 1.34 times as long as wide. Protibial spurs rounded at apex. Distal gonocoxite (Fig. 3A) rounded apically, densely covered with setae; spiculum ventrale as in Fig. 3B.

###### Diagnosis.

This new species is morphologically similar to *P.oblonga*, but can be distinguished from it by the following male character states: ventral surface of mesotarsomeres I–II with hair brushes, mesotarsomere III with small hairy tuft (ventral surface of mesotarsomeres I–III with hairy brush in *P.oblonga*); surface of elytra with dense, rather smooth punctation and regular wrinkle (surface of elytra with fine punctures and irregular wrinkles in *P.oblonga*); parameres strongly elongate, 3.0 times as long as wide (parameres elongate, 2.75 times as long as wide in *P.oblonga*).

###### Etymology.

This species is named for Banbar County, where the type locality is located.

###### Distribution.

Banbar County, Xizang, China.

##### ﻿ Pseudognaptorina himalayana

Taxon classificationAnimaliaColeopteraTenebrionidae

X.-M. Li sp. nov.

584201EB-90EF-5CD7-818D-FEB0E77574DB

https://zoobank.org/2AD0AE17-2347-47BA-94A7-1674F0633791

###### Type materials.

***Holotype***: China • ♂ (MHBU HBU(E)339877): Qucho Mountain, Lhunze County, Xizang/ 28°19.562' N, 92°19.112' E/ Alt. 4824 m/ 2019-VII-30/ Xiu-Min Li & Zhao Pan leg.

###### Description.

**Figure 5.**
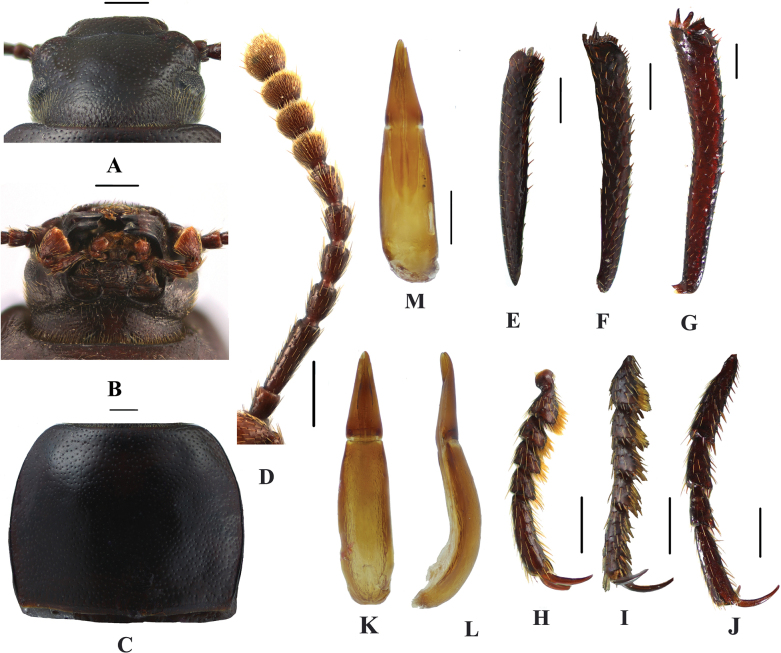
*Pseudognaptorinahimalayana* X.-M. Li, sp. nov., male **A** head, dorsal view **B** head, ventral view **C** pronotum **D** antenna **E** protibia **F** mesotibia **G** metatibia **H** protarsus **I** mesotarsus **J** metatarsus **K–M** aedeagus **K** dorsal view **L** lateral view **M** ventral view. Scale bars: 0.5 mm.

**Figure 6.**
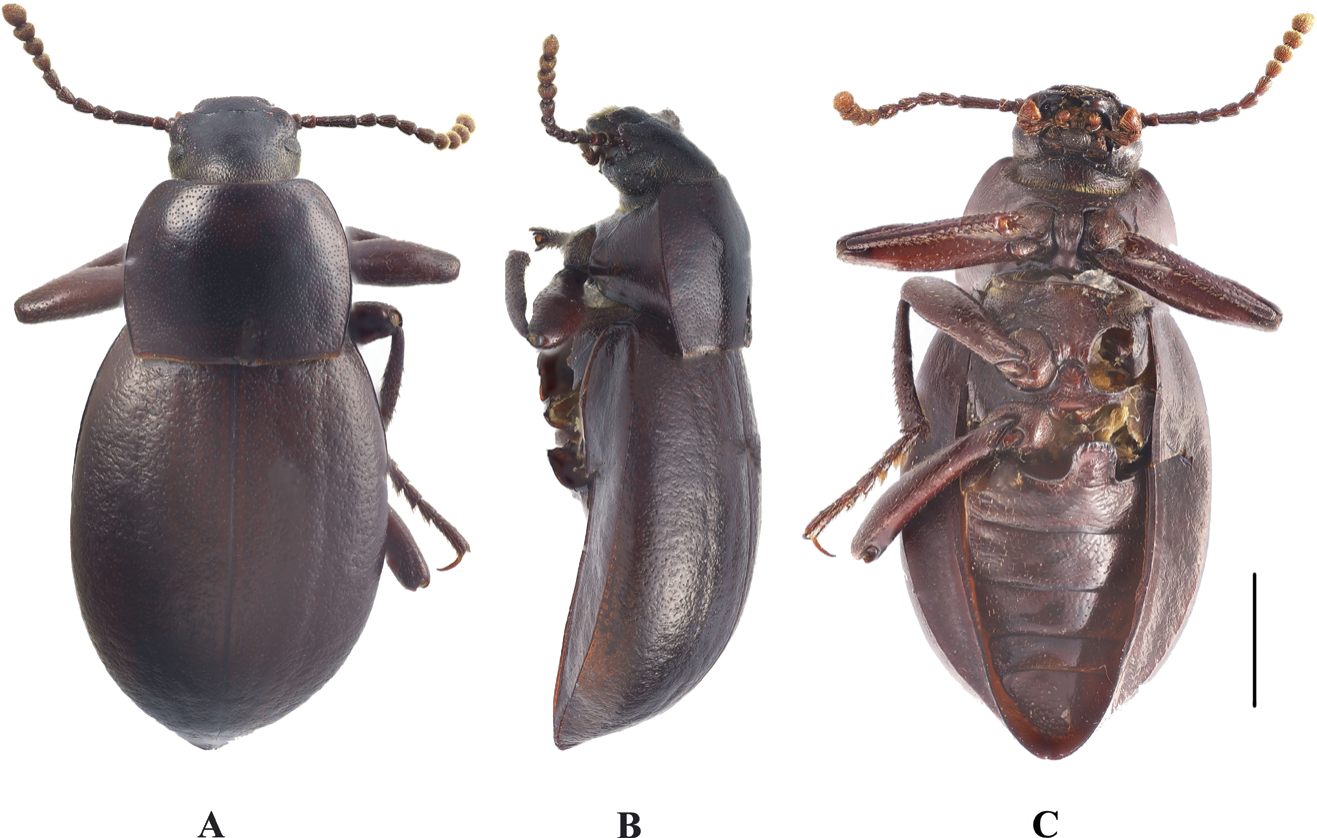
Habitus of *Pseudognaptorinahimalayana* X.-M. Li, sp. nov., male, holotype. **A** dorsal view **B** lateral view **C** ventral view. Scale bar: 2.0 mm.

**Male** (Figs 5, 6A–C). Body length 10.3 mm, width 4.9 mm; shiny, brownish; antennae, palpi, and tarsi brown.

***Head*** (Fig. 5A, B). Anterior margin of clypeus slightly sinuate. Head widest at eye level. Lateral margin of head with pair of projections between antennal base and oculus, brownish red. Genal margin arcuately converging before eyes. Eyes barely protruding beyond contour of head. Vertex flat or slightly convex, with uniform punctures. Antennae (Fig. 5D) slender, long, and reaching beyond pronotal base when posteriorly extended; antennomeres VIII–X oval, XI spindle-shaped. Length (width) ratio of antennomeres II–XI as follows: 11.0(10.0): 27.7(10.0): 12.5(10.0): 12.5(10.0): 12.5(10.0): 13.4(10.0): 10.4(12.5): 10.4(14.8): 10.4(15.6): 15.5(15.8).

***Prothorax*.** Pronotum (Fig. 5C) transverse, 1.29 times as wide as long, widest in middle, 1.72 times as wide as head. Ratio of width on anterior margin to its maximum width and posterior margin 0.58: 1.00: 0.92. Lateral margins of pronotum arcuately narrowing in middle, bordered along entire length; posterior margin straight; anterior margin slightly emarginate; anterior angles widely obtuse, posterior angles slightly obtuse. Surface of pronotum very narrowly flattened along lateral margins from base nearly to anterior angles, covered with dense punctation. Hypomera covered shallow longitudinal wrinkles and granules. Prosternum before procoxae gently sloping. Prosternal process gently sloping behind procoxae, forming obtuse projection.

***Pterothorax*.** Elytra oblong-oval and convex, 1.32–1.33 times as long as wide, 1.38–1.41 times as wide as pronotum, widest in apical third. Dorsal surface of elytra passing into outer (deflexed) surface without traces of humeral carina. Outer margin of epipleura visible in dorsal view at basal third and apex. Surface of elytra with dense, rather smooth punctation and wrinkles almost vanishing on apical declivity.

***Legs*** (Fig. 5E–J). Femora and tibiae moderately thickened. Ratio of length (width) of pro-, meso-, and metatibiae: 45.0(6.5): 50.0(7.5): 82.5(8.0). Protibiae narrow, straight with shorter spurs; mesotibiae slightly arcuately curved; metatibiae arcuately curved, narrow. Ventral surface of protarsomeres I–III with hairy brush; ventral surface of mesotarsomeres I–II with hairy brush. Ratio of length(width) of metatarsomeres I–IV: 32.0(9.5): 17.0(10.0): 13.5(9.0): 30.0(8.5).

***Abdomen*.** Abdominal ventrites rather sparsely covered with minute, pale, recumbent setae.

***Aedeagus*** (Fig. 5K–M). Length of aedeagus 2.30 mm, width 0.48 mm; length of parameres 0.83 mm, width 0.34 mm. Slightly curved to ventral side apically in lateral view. Parameres moderately elongate, regularly narrowing towards apex; outer margins slightly curved to ventral side apically in lateral view.

###### Diagnosis.

This new species is morphologically similar to *P.migana* but can be distinguished from it by the following male character states: pronotum 1.34 times as wide as long, lateral margins arcuately narrowed at basal 2/3 (pronotum 1.29 times as wide as long, lateral margins of pronotum regularly arcuate in *P.migana*); ventral surface of mesotarsomeres I–III with hair brushes (mesotarsomeres I–II with hair brushes, mesotarsomere III with small hairy tuft in *P.migana*); parameres moderately elongate, regularly narrowing towards apex, more obtuse from basal half to apex (parameres strongly elongate, widest at base, regularly narrowing towards apex, more acute from basal half to apex in *P.migana*).

###### Etymology.

This species is named for the Himalayas, where the type locality of the species is located.

###### Distribution.

Lhunze County, Xizang, China.

##### ﻿ Pseudognaptorina migana

Taxon classificationAnimaliaColeopteraTenebrionidae

X.-M. Li sp. nov.

894F98F7-50CC-5FA9-A623-5D3CC48509FE

https://zoobank.org/A3F1F50C-5F43-4F93-80B1-99960E9A47FA

###### Type materials.

***Holotype***: China • ♂ (MHBU HBU(E)339879): Miga Mountain pass, Gongbogyamda County, Xizang/ 29°84.105' N, 92°33.422' E/ Alt. 4775 m/ 2023-VII-18/ Xiu-Min Li & Tong-Yang Guo leg. ***Paratypes***: China • 10♂♂, 20♀♀ (MHBU HBU(E)339880–339909): same data as holotype; China • 1♂, 1♀ (MHBU HBU(E)339910–339911): Miga pass, Gongbogyamda County, Xizang/ 29°84.105' N, 92°33.422' E/ Alt. 4750 m/ 2019-VII-26/ Xiu-Min Li & Zhao Pan leg.

###### Description.

**Figure 7.**
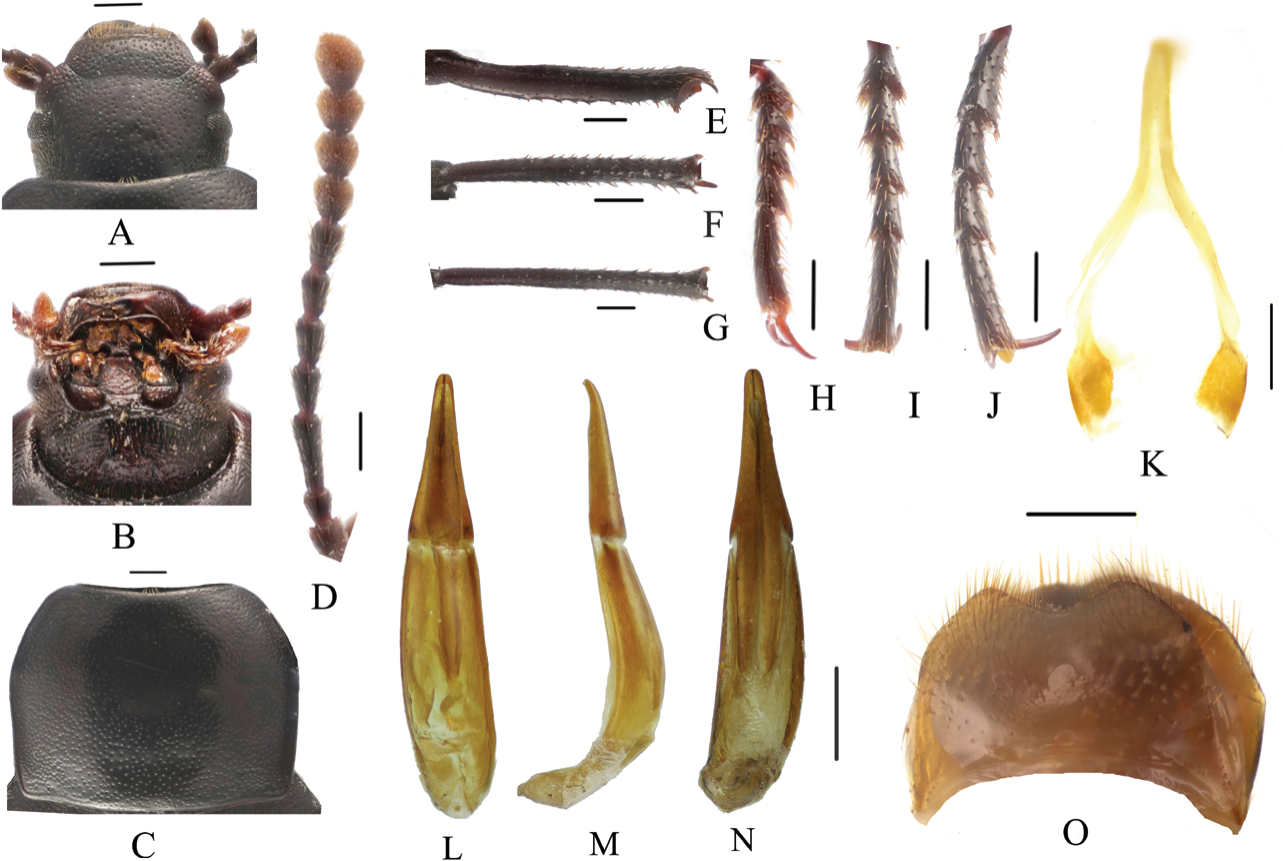
*Pseudognaptorinamigana* X.-M. Li, sp. nov., male **A** head, dorsal view **B** head, ventral view **C** pronotum **D** antenna **E** protibia **F** mesotibia **G** metatibia **H** protarsus **I** mesotarsus **J** metatarsus **K** spiculum gastrale **L–N** aedeagus **L** dorsal view **M** lateral view **N** ventral view **O** abdominal sternite VIII. Scale bars: 0.5 mm.

**Male** (Fig. 7, 8A–C). Body length 10.5–11.3 mm, width 4.9–5.2 mm; shiny, black or brownish; antennae, palpi, and tarsi brown.

**Figure 8.**
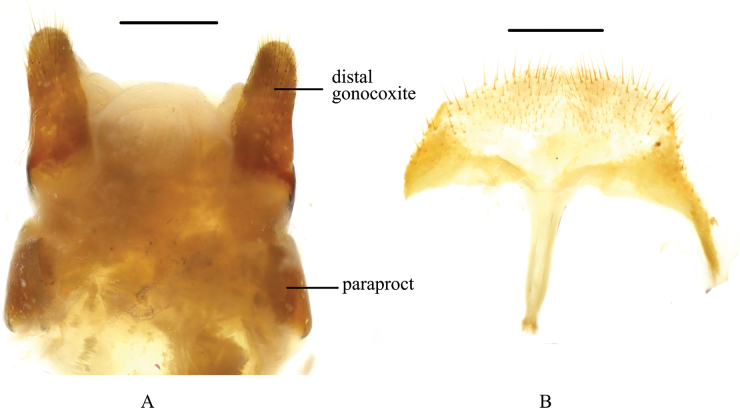
*Pseudognaptorinamigana* X.-M. Li, sp. nov., female **A** ovipositor **B** spiculum ventrale. Scale bars: 0.5 mm.

**Figure 9.**
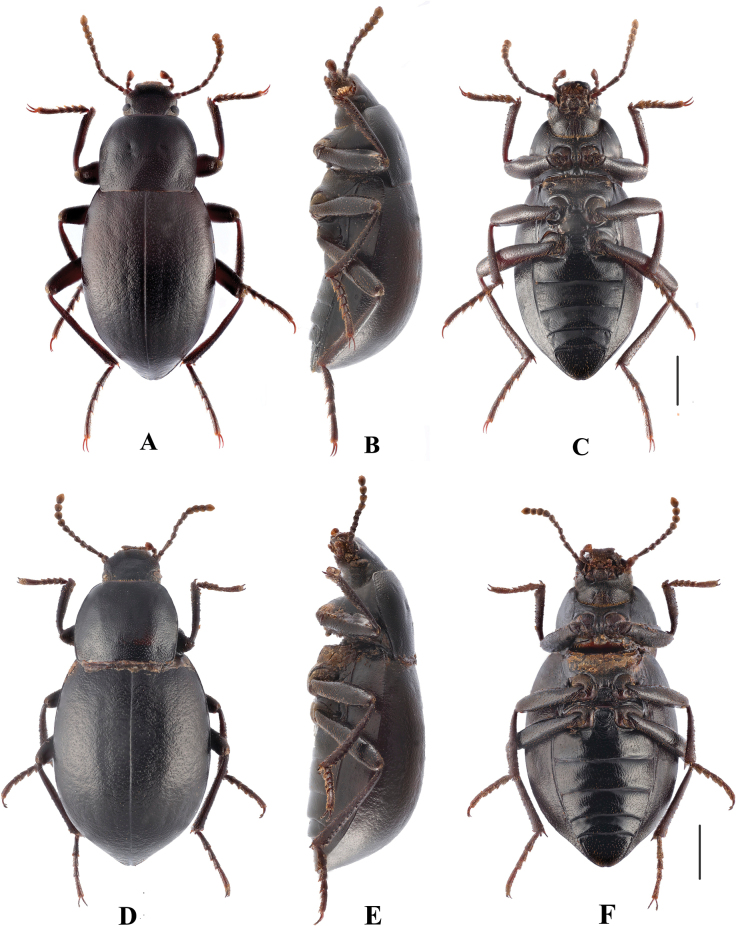
Habitus of *Pseudognaptorinamigana* X.-M. Li, sp. nov. **A–C** male, holotype **D–F** female, paratype **A, D** dorsal views **B, E** lateral views **C, F** ventral views. Scale bars: 2.0 mm.

***Head*** (Fig. 7A, B). Anterior margin of clypeus slightly sinuate. Head widest at eye level. Lateral margin of head with pair of projections between antennal base and oculus, brownish red. Genal margin arcuately converging before eyes. Eyes barely protruding beyond contour of head. Vertex flat or slightly convex, with uniform punctures. Antennae (Fig. 7D) slender and long, reaching pronotal base when posteriorly extended, antennomere III very long, 2.4 times as long as antennomeres II, antennomeres VIII–X oval, XI spindle-shaped. Length (width) ratio of antennomeres II–XI as follows: 11.8(10.0): 28.1(10.0): 14.5(10.0): 14.5(10.0): 14.5(10.0): 14.5(10.0): 13.3(12.0): 13.3(13.0): 13.3(14.0): 19.5(14.0).

***Prothorax*.** Pronotum (Fig. 7C) transverse, 1.34 times as wide as long, widest in middle, 1.71 times as wide as head. Ratio of width on anterior margin to its maximum width and posterior margin 0.52: 1.00: 0.92. Lateral margins of pronotum arcuately narrowing to anterior margin, bordered along entire length; posterior margin straight; anterior margin slightly emarginate; anterior angles widely, obtusely angled; posterior angles almost rectangular. Surface of pronotum slightly convex between lateral margins, covered with fine, dense punctation. Hypomera covered shallow, longitudinal wrinkles and granules. Prosternum before procoxae gently sloping. Prosternal process gently sloping behind procoxae, forming obtuse projection.

***Pterothorax*.** Elytra oblong-oval and convex, 1.38–1.40 times as long as wide, 1.35–1.37 times as wide as pronotum, widest in apical third. Dorsal surface of elytra passing into outer (deflexed) surface without traces of humeral carina. Outer margin of epipleura visible in dorsal view at basal third and apex. Surface of elytra with dense, rather smooth punctation and wrinkles almost vanishing on apical declivity.

***Legs*** (Fig. 7E–J). Femora and tibiae moderately thickened. Ratio of length (width) pro-, meso-, and metatibiae: 56.2(9.1): 55.8(9.5): 94.1(11.0). Protibiae straight with shorter spurs, inner surface of protibiae slightly widened at basal third; mesotibiae slightly arcuately curved; metatibiae arcuately curved, narrow. Ventral surface of protarsomeres I–III with hairy brush; ventral surface of mesotarsomeres I–II with hairy brush. Ratio of length (width) of metatarsomeres I–IV: 29.9(10.0): 19.5(10.0): 17.1(10.0): 29.4(7.9).

***Abdomen*.** Abdominal ventrites rather sparsely covered with minute, pale, recumbent setae.

***Aedeagus*** (Fig. 7K–O). Length of aedeagus 2.34 mm, width 0.46 mm; length of parameres 0.89 mm, width 0.35 mm. Slightly curved to ventral side apically in lateral view. Parameres strongly elongate, widest at base, regularly narrowing towards apex; outer margins slightly curved to ventral side apically in lateral view. Spiculum gastrale as in Fig. 7K. Posterior margin of abdominal sternite VIII sinuate (Fig. 7O).

**Female** (Figs 8A, B, 9D–F). Body larger and wider than male, length 12.1–12.7 mm, width 6.0–6.4 mm. Antennae shorter than male, not posteriorly reaching base of pronotum when posteriorly extended. Pronotum 1.5 times as wide as long, widest in middle, lateral margins subparallel from base to middle and narrowing toward anterior angles curved, sides of pronotum slightly convex; 1.73 times as wide as head; with very dense punctation. Elytra oval, more convex than male, 1.37 times as long as wide. Protibial spurs with small, pointed at apex. Distal gonocoxite (Fig. 8A) rounded apically, densely covered with setae; spiculum ventrale as in Fig. 8B.

###### Diagnosis.

This new species is morphologically similar to *P.himalayana*, but can be distinguished from it by the following male character states: pronotum 1.29 times as wide as long, lateral margins of pronotum regularly arcuate (pronotum 1.34 times as wide as long, lateral margins arcuately narrowed at basal 2/3 in *P.himalayana*); mesotarsomeres I–II with hair brushes, mesotarsomere III with small hairy tuft (ventral surface of mesotarsomeres I–III with hair brushes in *P.himalayana*); parameres strongly elongate, widest at base, regularly narrowing towards apex, more acute from basal half to apex (parameres moderately elongate, regularly narrowing towards apex, more obtuse from basal half to apex in *P.himalayana*).

###### Etymology.

This species is named after the type locality, Miga Pass.

###### Distribution.

Gongbogyamda County, Xizang, China.

##### ﻿ Pseudognaptorina oblonga

Taxon classificationAnimaliaColeopteraTenebrionidae

X.-M. Li sp. nov.

562B8276-9134-5636-B165-FE3E69513446

https://zoobank.org/460ECFD4-85F7-410A-9D6D-D74A2A6DD9CD

###### Type materials.

***Holotype***: China • ♂ (MHBU HBU(E)339912): Lhari County, Xizang / 2013-VII-22/ Xing-Long Bai & Jun-Sheng Shan leg. ***Paratypes***: China • 2♂♂, 4♀♀ (MHBU HBU(E)339913–339918): same data as holotype; China • 4♂♂, 5♀♀ (MHBU HBU(E)339919–339927): Arza Township, Lhari County, Xizang/ 30°37.104'N, 93°24.307'E/ Alt. 4300 m/ 2019-VIII-9/ Guo-Dong Ren, Ya-Lin Li & Xing-Long Bai leg.; China • 1♂ (MHBU HBU(E)339928): Lhari County, Xizang/ 30°45.225'N, 93°13.162'E/ Alt. 4762 m/ 2023-VII-17/ Xiu-Min Li & Tong-Yang Guo leg.

###### Description.

**Figure 10.**
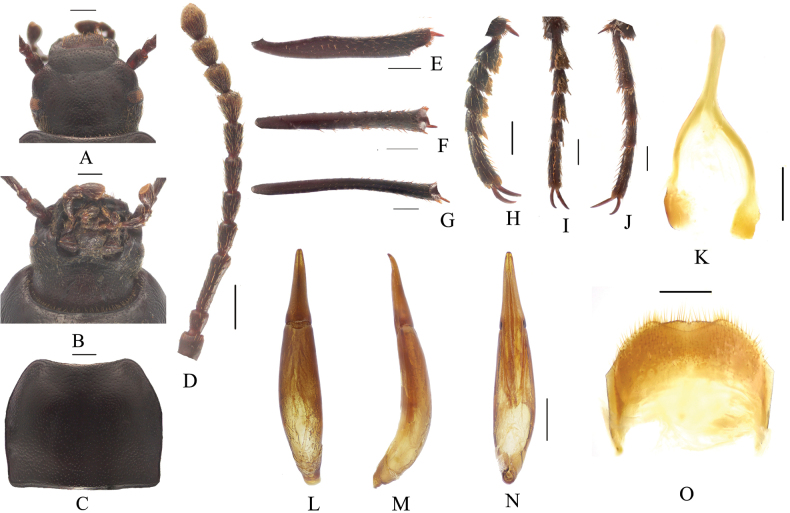
*Pseudognaptorinaoblonga* X.-M. Li, sp. nov., male **A** head, dorsal view **B** head, ventral view **C** pronotum **D** antenna **E** protibia **F** mesotibia **G** metatibia **H** protarsus **I** mesotarsus **J** metatarsus **K** spiculum gastrale **L–N** aedeagus **L** dorsal view **M** lateral view **N** ventral view **O** abdominal sternite VIII. Scale bars: 0.5 mm.

**Figure 11.**
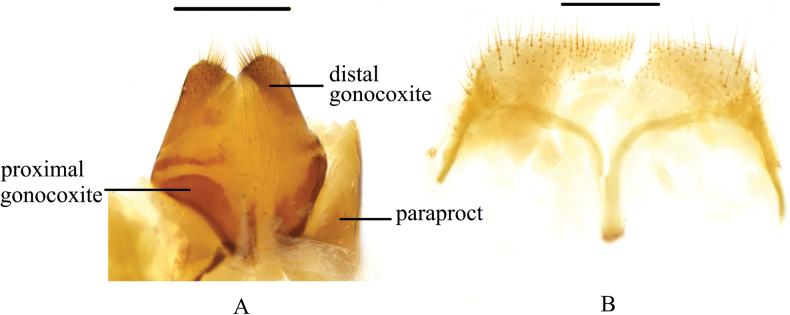
*Pseudognaptorinaoblonga* X.-M. Li, sp. nov., female. **A** ovipositor **B** spiculum ventrale. Scale bars: 0.5 mm.

**Male** (Figs 10, 11A–C). Body length 10.8–11.3 mm, width 5.0–5.1 mm; shiny, black or brownish; antennae, palpi, and tarsi brown.

**Figure 12.**
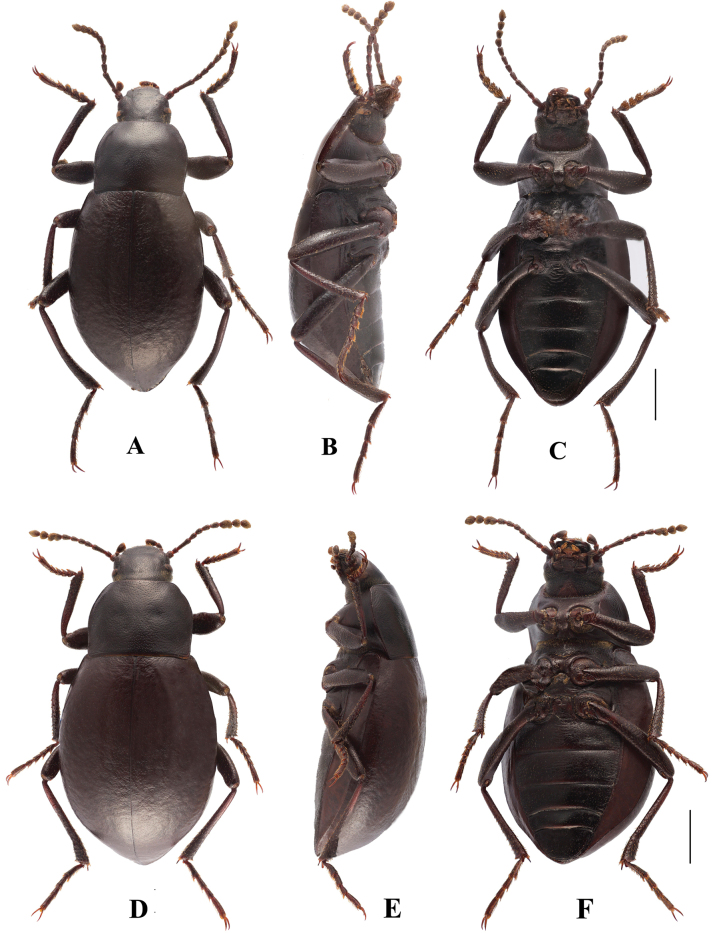
Habitus of *Pseudognaptorinaoblonga* X.-M. Li, sp. nov. **A–C** male, holotype **D–F** female, paratype **A, D** dorsal views **B, E** lateral views **C, F** ventral views. Scale bars: 2.0 mm.

***Head*** (Fig. 10A, B). Anterior margin of clypeus slightly sinuate. Head widest at eye level. Lateral margin of head with pair of projections between antennal base and oculus, brownish red. Genal margin arcuately converging before eyes. Eyes barely protruding beyond contour of head. Vertex flat or slightly convex, with uniform punctures. Antennae (Fig. 10D) slender and long, reaching beyond pronotal base when posteriorly extended, antennomere III very long, 3.1 times as long as antennomere II, antennomeres VIII–X oval, XI spindle-shaped. Length (width) ratio of antennomeres II–XI as follows: 10.0(10.0): 31.3(10.0): 15.9(10.0): 15.1(10.0): 16.2(10.0): 16.2(10.3): 15.3(12.5): 15.3(12.5): 14.8(12.5): 19.8(13.8).

***Prothorax*.** Pronotum (Fig. 10C) transverse, 1.40 times as wide as long, widest in middle, 1.67 times as wide as head. Ratio of width on anterior margin to its maximum width and posterior margin 0.57: 1.00: 0.96. Lateral margins of pronotum arcuately narrowing to anterior margin, bordered along entire length; posterior margin straight; anterior margin slightly emarginate; anterior angles widely, obtusely angled; posterior angles almost rectangular. Surface of pronotum very narrowly flattened along lateral margins from base nearly to anterior angles, covered with dense punctation. Hypomera covered shallow longitudinal wrinkles and granules. Prosternum before procoxae gently sloping. Prosternal process gently sloping behind procoxae, forming obtuse projection.

***Pterothorax*.** Elytra oblong-oval and convex, 1.45–1.49 times as long as wide, 1.44–1.46 times as wide as pronotum, widest at apical third. Dorsal surface of elytra passing into outer (deflexed) surface without traces of humeral carina. Outer margin of epipleura visible in dorsal view at basal third and apex. Surface of elytra with dense, rather smooth punctation and wrinkle almost vanishing on apical declivity.

***Legs*** (Fig. 10E–J). Femora and tibiae moderately thickened. Ratio of length(width) of pro-, meso-, and metatibiae: 46.8(6.5): 64.6(7.5): 94.1(8.0). Protibiae straight with shorter spur, inner surface of protibiae slightly widen in basal 1/3; mesotibiae slightly arcuately curved; metatibiae arcuately curved, narrow. Ventral surface of protarsomeres I–III with hairy brush; ventral surface of mesotarsomere I–III with hairy brush. Ratio of length(width) of metatarsomeres I–IV segments:27.5(9.9): 25.0(9.1): 17.5(8.6): 37.5(9.3).

***Abdomen*.** Abdominal ventrites rather sparsely covered with minute, pale, recumbent setae.

***Aedeagus*** (Fig. 10K–O). Length of aedeagus 2.77 mm, width 0.53 mm; length of parameres 0.88 mm, width 0.32 mm. Slightly curved to ventral side apically in lateral view. Parameres strongly elongate, widest at base, regularly narrowing towards apex; outer margins slightly curved to ventral side apically in lateral view. Spiculum gastrale as in Fig. 10K. Posterior margin of abdominal sternite VIII sinuate (Fig. 10O).

**Female** (Fig. 11A, B, 12D–F). Body larger and wider than male, length 12.1–12.5 mm, width 5.9–6.1 mm. Outer margin of head above base of antennae with widely, obtusely angled emargination, less sharp than in male. Antennae shorter than in male, not posteriorly reaching base of pronotum when posteriorly extended. Pronotum 1.45 times as wide as long, widest in middle, lateral margins subparallel from base to middle and narrowing toward anterior angles arcuately, sides of pronotum slightly convex; 1.72 times as wide as head; with very dense punctation. Elytra oval, more convex than male, 1.32 times as long as wide. Protibial spurs small, pointed at apex. Distal gonocoxite (Fig. 11A) rounded apically, densely covered with setae; spiculum ventrale as in Fig. 11B.

###### Diagnosis.

This new species is morphologically similar to *P.banbarica*, but can be distinguished from it by the following male character states: ventral surface of mesotarsomeres I–III with hairy brush (ventral surface of mesotarsomeres I–II with hair brushes, mesotarsomere III with small hairy tuft in *P.banbarica*); surface of elytra with fine punctures and irregular wrinkles (surface of elytra with dense, rather smooth punctation and wrinkle in *P.banbarica*).

###### Etymology.

This species is named from the Latin adjective “oblonga”, in reference to its elongate aedeagus.

###### Distribution.

Lhari County, Xizang, China.

##### ﻿ Pseudognaptorina rectangularis

Taxon classificationAnimaliaColeopteraTenebrionidae

X.-M. Li sp. nov.

816F668F-F9C9-59E4-8B8D-F7345A4780E0

https://zoobank.org/2AF10B93-D9D1-4DE2-AEA5-480ABF9D4098

###### Type materials.

***Holotype***: China • ♂ (MHBU HBU(E)339929): Hongyuan County, Sichuan/ 32°01.96'N, 102°01.99'E/ Alt. 3451 m/ 2021-VII-20/ Xiu-Min Li leg.

###### Description.

**Figure 13.**
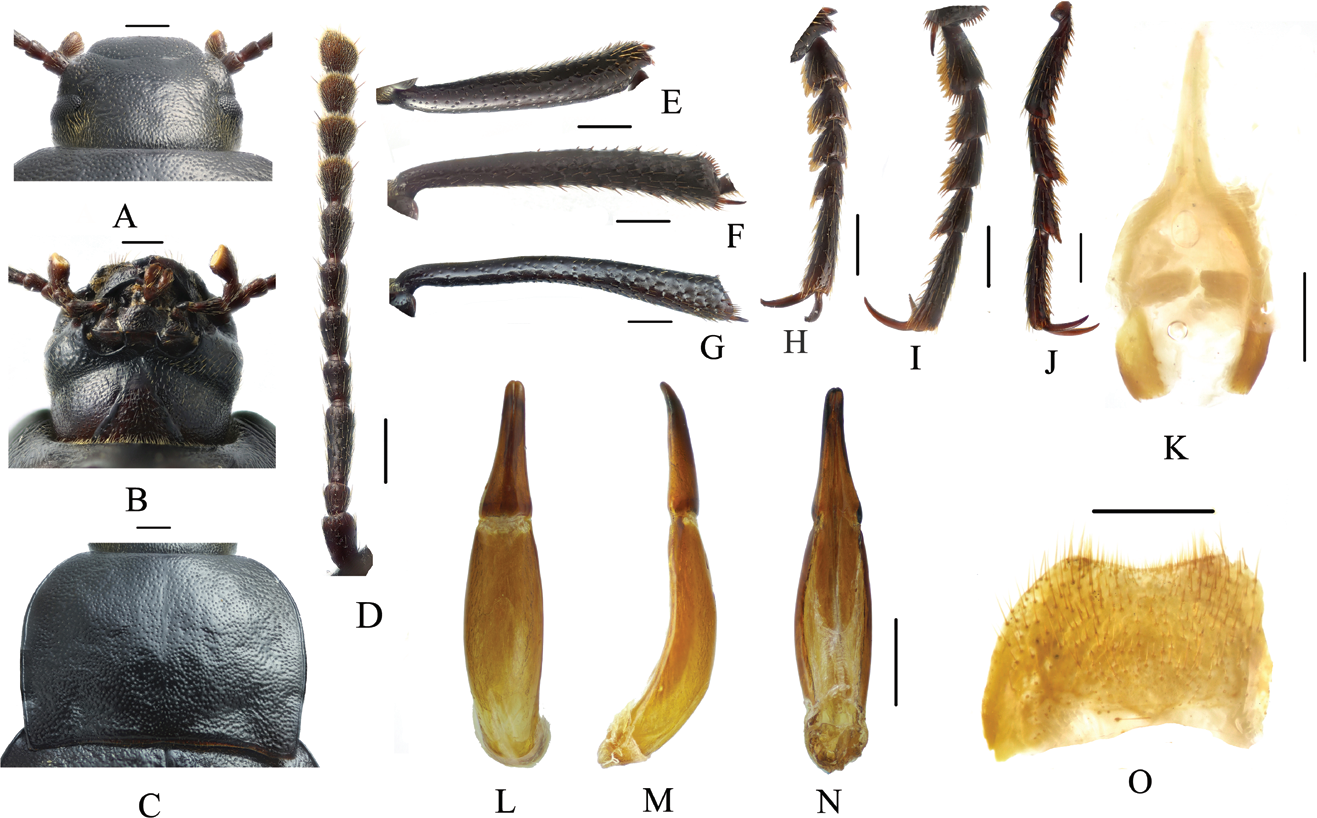
*Pseudognaptorinarectangularis* X.-M. Li, sp. nov., male **A** head, dorsal view **B** head, ventral view **C** pronotum **D** antenna **E** protibia **F** mesotibia **G** metatibia **H** protarsus **I** mesotarsus **J** metatarsus **K** spiculum gastrale **L–N** aedeagus **L** dorsal view **M** lateral view **N** ventral view **O** abdominal sternite VIII. Scale bars: 0.5 mm.

**Figure 14.**
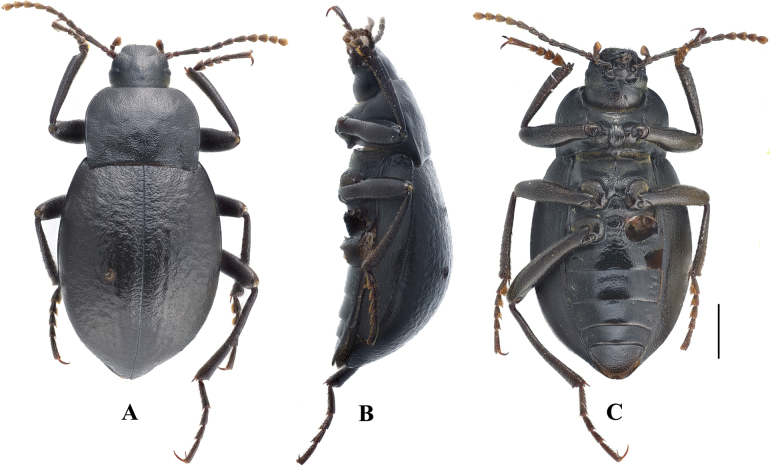
Habitus of *Pseudognaptorinarectangularis* X.-M. Li, sp. nov., male, holotype **A** dorsal view **B** lateral view **C** ventral view. Scale bar: 2.0 mm.

**Male** (Figs 13, 14A–C). Body length 12.6 mm, width 6.2 mm; shiny, black; antennae, palpi, and tarsi brown.

***Head*** (Fig. 13A, B). Anterior margin of clypeus slightly sinuate. Head widest at eye level. Lateral margin of head with pair of projections between antennal base and oculus, brownish red. Genal margin arcuately converging before eyes. Eyes barely protruding beyond contour of head. Vertex flat or slightly convex, with uniform punctures. Antennae (Fig. 13D) slender, long, and reaching pronotal base when posteriorly extended; antennomere III very long, 2.9 times as long as antennomere II; antennomeres VIII–X oval; XI spindle-shaped. Length (width) ratio of antennomeres II–XI as follows: 10.9(9.4): 27.1(10.7): 14.8(10.0): 16.2(10.0): 15.5(10.0): 18.9(10.0): 15.1(11.5): 13.9(13.3): 13.9(13.3): 15.2(13.9).

***Prothorax*.** Pronotum (Fig. 13C) transverse, 1.52 times as wide as long, widest in middle, 1.84 times as wide as head. Ratio of width on anterior margin to its maximum width and posterior margin 0.55: 1.00: 0.97. Lateral margins of pronotum arcuately narrowing to anterior margin, bordered along entire length; posterior margin straight; anterior margin slightly emarginate; anterior angles widely, obtusely angled; posterior angles almost rectangular. Surface of pronotum very narrowly flattened along lateral margins from base nearly to anterior angles, covered with dense punctation. Hypomera covered shallow longitudinal wrinkles and granules; inner part covered with longitudinal rugae. Prosternum gently sloping before procoxae. Prosternal process gently sloping behind procoxae, forming obtuse projection.

***Pterothorax*.** Elytra oblong-oval and convex, 1.31 times as long as wide, 1.43 times as wide as pronotum, widest at apical third. Dorsal surface of elytra passing into outer (deflexed) surface without traces of humeral carina. Outer margin of epipleura visible in dorsal view at basal third and apex. Surface of elytra with dense, rather smooth punctation and wrinkles almost vanishing on apical declivity.

***Legs*** (Fig. 13E–J). Femora and tibiae moderately thickened. Ratio of length (width) of pro-, meso-, and metatibiae: 57.2(9.2): 59.1(9.4): 77.9(10.3). Protibiae straight with shorter spur, inner surface of protibiae nearly flat; mesotibiae slightly curved; metatibiae slightly curved. Ventral surface of protarsomeres I–III with hairy brush; ventral surface of mesotarsomeres I–II with hairy brush. Ratio of length(width) of metatarsomeres I–IV: 34.5(9.7): 22.5(10.0): 22.8(10.0): 31.9(7.7).

***Aedeagus*** (Fig. 13K–O). Length of aedeagus 2.18 mm, width 0.46 mm; length of parameres 0.79 mm, width 0.31 mm. Slightly curved to ventral side apically in lateral view. Parameres strongly elongate, widest at base, regularly narrowing towards apex; outer margins slightly curved to ventral side apically in lateral view. Spiculum gastrale as in Fig. 13K. Posterior margin of abdominal sternite VIII sinuate (Fig. 13O).

###### Diagnosis.

This new species is morphologically similar to *P.flata*, but can be distinguished from it by the following male character states: pronotum transverse, 1.52 times as wide as long, surface flatted, posterior angles nearly rectangular (pronotum transverse, 1.36 times as wide as long, surface explanate and slightly concave, posterior angles obtusely rounded in *P.flata*); ventral surface of mesotarsomeres I–II with hair brushes, mesotarsomere III with small hairy tuft (ventral surface of mesotarsomeres I–III with hair brushes in *P.flata*).

###### Etymology.

This species is named from the Latin adjective “*rectangularis*”, in reference to its sub-rectangular prothorax.

###### Distribution.

Hongyuan County, Sichuan, China.

##### ﻿ Pseudognaptorina reni

Taxon classificationAnimaliaColeopteraTenebrionidae

X.-M. Li sp. nov.

3838EE23-4D57-5E72-AD22-6A6671BB5D25

https://zoobank.org/62A03392-4ADE-414F-9E21-00E41A071DAA

###### Type materials.

***Holotype***: China • ♂ (MHBU HBU(E)339930): Vajra Mountain pass, Zadowa County, Qinghai/ 32°47.98' N, 95°09.26' E/ Alt. 4718 m/ 2019-VII-26/ Guo-Dong Ren & Yi-Ping Niu leg. ***Paratypes***: China • 10♂♂, 20♀♀ (MHBU HBU(E)339931–339960): same data as holotype; China • 3♂♂, 10♀♀ (MHBU HBU(E)339961–339973): Konge township, Bachen County, Xizang/ 32°31.50' N, 94°43.31' E/ Alt. 4556 m/ 2022-VII-4/ Guo-Dong Ren & Yi-Ping Niu leg.

###### Description.

**Figure 15.**
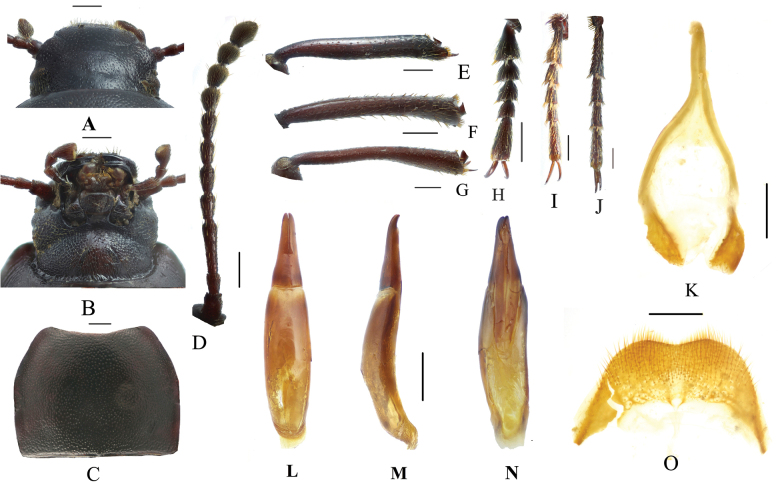
*Pseudognaptorinareni* X.-M. Li, sp. nov., male **A** head, dorsal view **B** head, ventral view **C** pronotum **D** antenna **E** protibia **F** mesotibia **G** metatibia **H** protarsus **I** mesotarsus **J** metatarsus **K** spiculum gastrale **L–N** aedeagus **L** dorsal view **M** lateral view **N** ventral view **O** abdominal sternite VIII. Scale bars: 0.5 mm.

**Figure 16.**
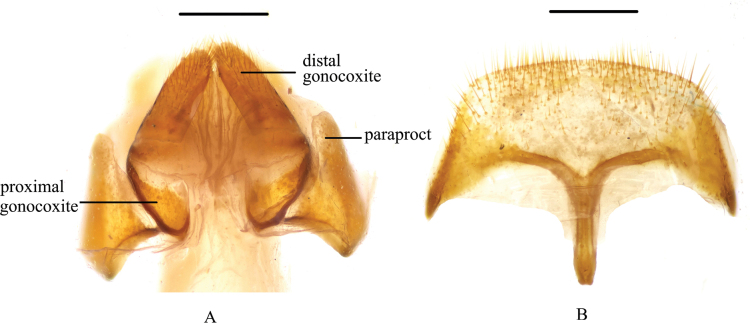
*Pseudognaptorinareni* X.-M. Li, sp. nov., female. **A** ovipositor **B** spiculum ventrale. Scale bars: 0.5 mm.

**Male** (Figs 15, 16A–C). Body length 13.6–14.1 mm, width 6.30–6.58 mm; black, slightly shiny, oval-oblong.

**Figure 17.**
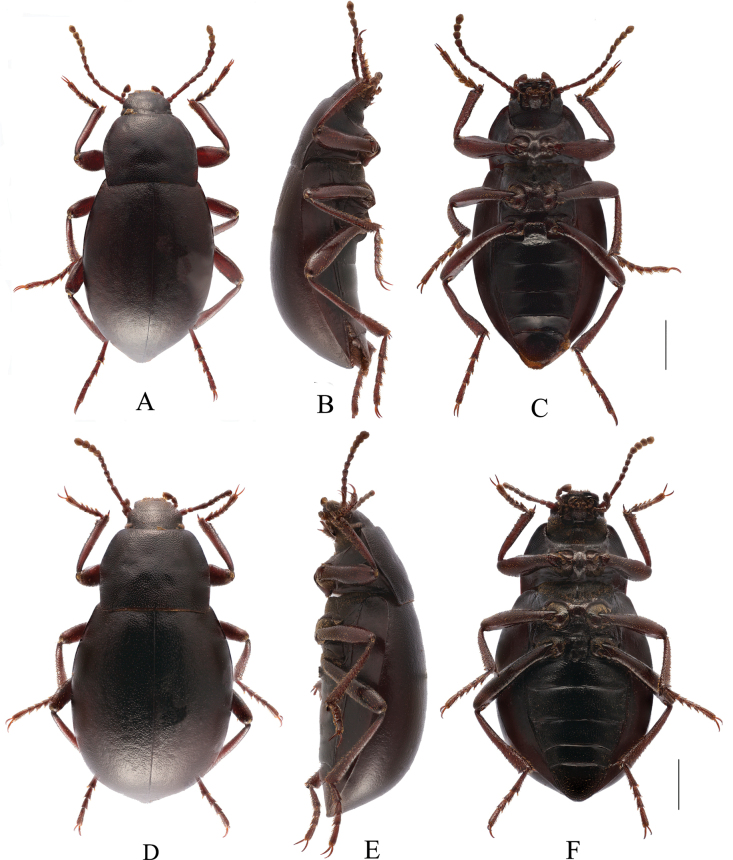
Habitus of *Pseudognaptorinareni* X.-M. Li, sp. nov. **A–C** male, holotype **D–F** female, paratype **A, D** dorsal views **B, E** lateral views **C, F** ventral views. Scale bar: 2.0 mm.

***Head*** (Fig. 15A, B). Anterior margin of clypeus slightly sinuate. Head widest at eye level. Lateral margin of head with pair of projections between antennal base and oculus, brownish red. Genal margin arcuately converging before eyes. Eyes barely protruding beyond contour of head. Vertex flat or slightly convex, with uniform punctures. Antennae (Fig. 15D) slender and long, reaching pronotal base when posteriorly extended, antennomere III very long, 2.7 times as long as antennomere II, antennomeres VIII–X oval, XI spindle-shaped. Length (width) ratio of antennomeres II–XI as follows: 12.5(10.0): 33.4(10.0): 16.6(10.0): 15.7(10.0): 14.8(10.0): 16.4(10.0): 14.1(12.9): 14.1(14.1): 14.1(14.7): 18.8(15.5).

***Prothorax*.** Pronotum (Fig. 15C) transverse, 1.50 times as wide as long, widest in middle, 1.72 times as wide as head. Ratio of width on anterior margin to its maximum width and posterior margin 0.54: 1.00: 0.95. Lateral margins of pronotum arcuately narrowing to anterior margin, bordered along entire length; posterior margin straight; anterior margin slightly emarginate; anterior angles widely, obtusely angled; posterior angles almost rectangular. Surface of pronotum slightly convex between lateral margins, very narrowly flattened along lateral margins from base nearly to anterior angles, covered with fine, dense punctation. Hypomera covered shallow, longitudinal wrinkles and granules. Prosternum before procoxae gently sloping. Prosternal process gently sloping behind procoxae, forming obtuse projection.

***Pterothorax*.** Elytra oblong-oval and convex, 1.43–1.48 times as long as wide, 1.39–1.41 times as wide as pronotum, widest at apical third. Dorsal surface of elytra passing into outer (deflexed) surface without traces of humeral carina. Outer margin of epipleura visible in dorsal view at basal third and apex. Surface of elytra with dense, rather smooth punctation and wrinkles almost vanishing on apical declivity.

***Legs*** (Fig. 15E–J). Femora and tibiae moderately thickened. Ratio of length (width) of pro-, meso-, and metatibiae: 56.0(8.6): 49.6(7.6): 68.0(8.6). Protibiae straight with shorter spur, inner surface of protibiae slightly widened at basal third; mesotibiae slightly arcuately curved; metatibiae curved, narrow. Ventral surface of protarsomeres I–III with hairy brush; ventral surface of mesotarsomeres I–II with hairy brush. Ratio of length (width) of metatarsomeres I–IV: 38.7(11.5): 21.1(10.0): 19.6(10.0): 31.1(10.0).

***Abdomen*.** Abdominal ventrites rather sparsely covered with minute, pale, recumbent setae.

***Aedeagus*** (Fig. 15K–O). Length of aedeagus 2.31 mm, width 0.51 mm; length of parameres 0.76 mm, width 0.33 mm. Slightly curved to ventral side apically in lateral view. Parameres strongly elongate, widest at base, regularly narrowing towards apex; outer margins slightly curved to ventral side apically in lateral view. Spiculum gastrale as in Fig. 15K. Posterior margin of abdominal sternite VIII sinuate (Fig. 15O).

**Female** (Figs 16A, B, 17D–F). Body larger and wider than male, length 14.1–15.0 mm, width 7.0–7.7 mm. Antennae shorter than in male, reaching base of pronotum when posteriorly extended. Pronotum 1.56 times as wide as long, widest in middle, lateral margins subparallel from base to middle and arcuately narrowing toward anterior angles, sides of pronotum slightly convex; 1.83 times as wide as head, with very dense punctation. Elytra oval, more convex than in male, 1.37 times as long as wide. Protibial spurs small, pointed at apex. Distal gonocoxite (Fig. 16A) rounded apically, densely covered with setae; spiculum ventrale as in Fig. 16B.

###### Diagnosis.

This new species is morphologically similar to *P.exsertogena*, but can be distinguished from it by the following male character states: pronotum 1.50 times as wide as long (pronotum 1.28 times as wide as long in *P.exsertogena*); surface of elytra with fine punctures and without regular wrinkles (surface of elytra with fine punctures and irregular wrinkles in *P.exsertogena*).

###### Etymology.

This species is named after Prof. Guo-Dong Ren, in recognition to his contributions in collecting specimens of *Pseudognaptorina*.

###### Distribution.

Zadowa and Bachen Counties, Xizang, China.

### ﻿Phylogenetic relationships

**Figure 18.**
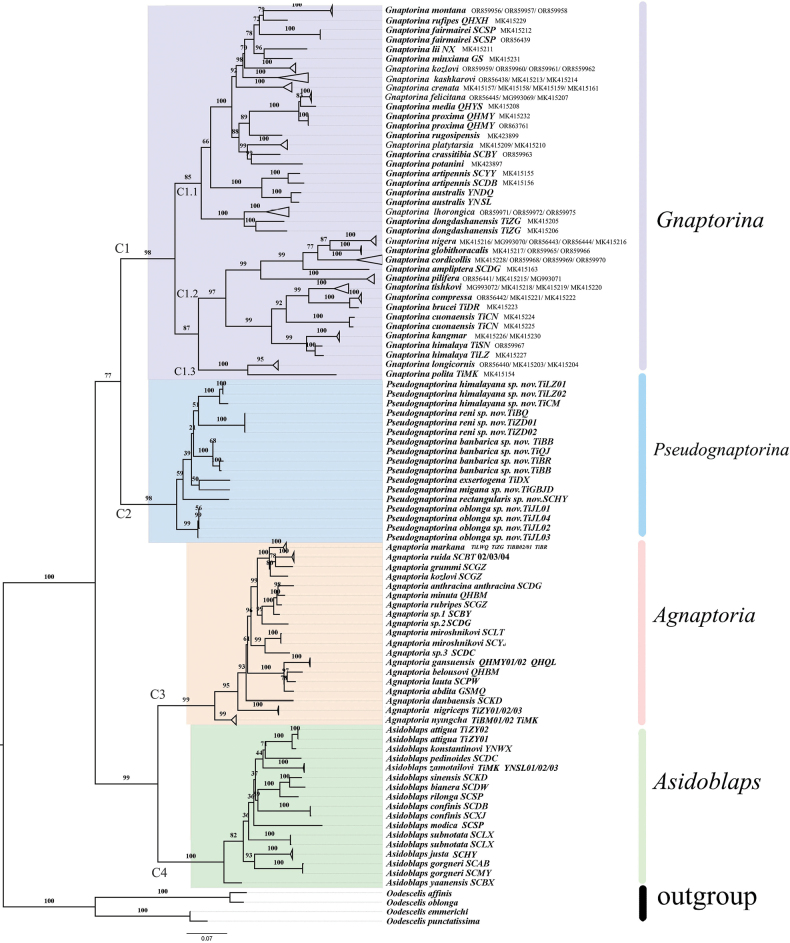
Maximum-likelihood phylogenetic tree based on mitochondrial COI gene sequences within four genera of the subtribe Gnaptorinina. Support for each node is represented by ultrafast bootstrap values (uBV).

The preliminary phylogenetic relationships were hypothesized from 147 samples of four genera (*Agnaptoria*, 31 samples; *Asidoblaps*, 22 samples; *Pseudognaptorina*, 17 samples; *Gnaptorina*, 77 samples) (Fig. 18). The ML tree exhibited a satisfactory correlation between these major clades and the current four genera. The individuals were grouped into four well-supported clades: clade C1 (*Gnaptorina*, uBV = 98), clade C2 (*Pseudognaptorina*, uBV = 98), clade C3 (*Agnaptoria*, uBV = 99), and clade C4 (*Asidoblaps*, uBV = 100).

### ﻿Geographical distribution and bionomics

**Figure 19.**
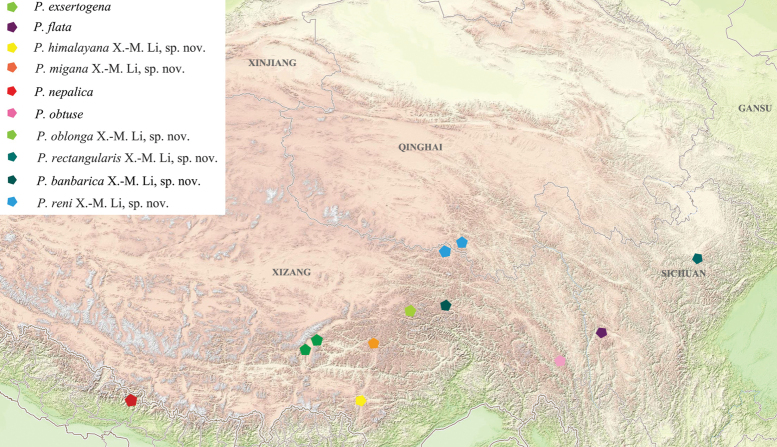
Geographical distribution of ten *Pseudognaptorina* species in this study.

Species of *Pseudognaptorina* exhibit distinctive distribution patterns within the geographical range of the genus. The genus has a wide distribution, mainly in Xizang, Sichuan, and Qinghai, China, except *P.nepalica* occurring in Nepal (Fig. 19).

**Figure 20.**
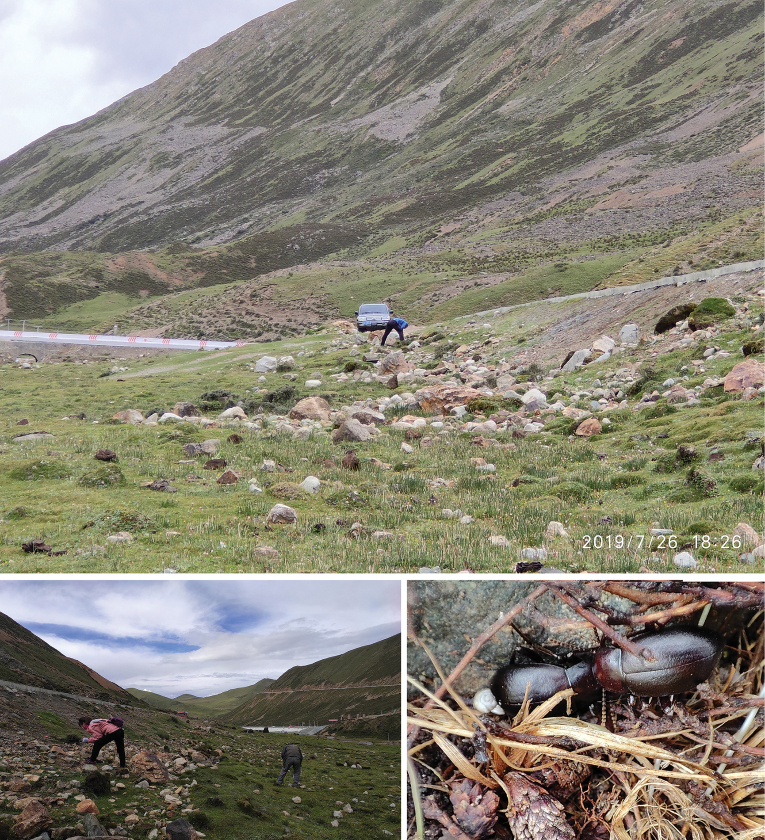
Habitat for *P.migana* X.-M. Li, sp. nov. Photographed by Xiu-Min Li, at Miga Mountain pass, Gongbogyamda County, Xizang, China, on June 26, 2019 and July 18, 2023.

All *Pseudognaptorina* species were found on the Qinghai-Xizang Plateau. Interestingly, these species have a narrow range on hillsides in subhumid environments. They were often found around the roots of underbrush and under stones or clods, and they probably feed on decaying plant roots or leaves (Fig. 20).

## ﻿Discussion

Gnaptorinina is a species-rich subtribe of Blaptini consisting of 189 species in 12 genera. These species are primarily distributed in deserts, semi-deserts, grasslands, meadows, woodlands, and high-elevation environments across the Qinghai-Xizang Plateau. We constructed the phylogenetic relationships of four genera within the subtribe Gnaptorinina based on COI gene sequences. Our analyses provide the first phylogenetic tree for the genus *Pseudognaptorina*, which is confirmed to be monophyletic. The distribution range of all available specimens reveals that four known species and six new species are widely and continuously distributed across the Qinghai-Xizang Plateau. Their habitat differs significantly from that of *Gnaptorina* and *Agnaptoria*. *Gnaptorina* and *Agnaptoria* are mainly distributed in the high-elevation areas with relatively arid environments (Shi et al. 2005; Li et al. 2021), whereas *Pseudognaptorina* primarily inhabits more humid valley environments traversed by the Yarlung Tsangpo, Lancang, and Nujiang rivers. We hypothesize that these species dispersed along river systems and formed their current geographical distribution patterns during the uplift of the Qinghai-Xizang Plateau. What is the relationship between species dispersal and the uplift of the Qinghai-Xizang Plateau? We will provide a comprehensive analysis and discussion of this issue in a forthcoming paper based on genomic data.

## Supplementary Material

XML Treatment for Pseudognaptorina

XML Treatment for Pseudognaptorina exsertogena

XML Treatment for Pseudognaptorina flata

XML Treatment for Pseudognaptorina nepalica

XML Treatment for Pseudognaptorina obtusa

XML Treatment for Pseudognaptorina banbarica

XML Treatment for Pseudognaptorina himalayana

XML Treatment for Pseudognaptorina migana

XML Treatment for Pseudognaptorina oblonga

XML Treatment for Pseudognaptorina rectangularis

XML Treatment for Pseudognaptorina reni

## ﻿Additional information

### Author ORCIDs

Xiu-Min Li https://orcid.org/0000-0003-0575-1869

Bao-Yue Zhang https://orcid.org/0009-0008-9251-4792

Ji-Gang Li https://orcid.org/0000-0001-8662-0734

Zhao Pan https://orcid.org/0000-0001-7798-0009

### Data availability

All data that support the findings of this study are available in the Supplementary file.

## Supplementary materials

Supplementary material 1 List of specimens Data type doc Explanation note List of specimens used in this study with the corresponding accession number. https://binary.pensoft.net/file/1302103This dataset is made available under the Open Database License (http://opendatacommons.org/licenses/odbl/1.0/). The Open Database License (ODbL) is a license agreement intended to allow users to freely share, modify, and use this Dataset while maintaining this same freedom for others, provided that the original source and author(s) are credited.Author: Xiu-Min Li
