# Supplementary material for: ﻿Systematic review of the genus Pseudognaptorina Kaszab, 1977 (Coleoptera, Tenebrionidae, Blaptinae, Blaptini) from the Qinghai-Xizang Plateau, with description of six new species
Source: Zookeys. 2025 Apr 8;1234:19–46. doi: 10.3897/zookeys.1234.137739 (PMC12000813; doi:10.3897/zookeys.1234.137739)
Supplement: Supplementary material 1 — List of specimens [file zookeys-1234-019_article-137739__-s001.doc]

**Table S1.** List of specimens used in this study with the corresponding accession number.

| No. | Species / Subspecies | Sampling locality | Elevation (m) | Date of collection | Collectors | Accession numbers (*COI*) |
| --- | --- | --- | --- | --- | --- | --- |
| 1 | *Pseudognaptorina banbarica* **sp. nov.** | Marxog Township, Banbar County, Xizang, China (TiBB01) | 4400 | 31.Ⅶ.2019 | G.-D. Ren *et al*. | PV197102 |
| 2 | *Pseudognaptorina banbarica* **sp. nov.** | Liangqu Township, Biru County, Xizang, China (TiBR) | 4063 | 10.Ⅶ.2023 | X.-M. Li *et al*. | PV197103 |
| 3 | *Pseudognaptorina banbarica* **sp. nov.** | Marxog Township, Banbar County, Xizang, China (TiBB02) | 4570 | 15.Ⅶ.2023 | X.-M. Li *et al*. | PV197104 |
| 4 | *Pseudognaptorina banbarica* **sp. nov.** | Zeyu Township, Qiongjie County, Xizang, China (TiQJ) | 3595 | 31.Ⅶ.2019 | G.-D. Ren *et al*. | PV197101 |
| 5 | *Pseudognaptorina exsertogena* | Xoggu Mountain, Damxung County, Xizang, China (TiDX) | 5147 | 9.VIII.2019 | X.-M. Li *et al*. | PV197108 |
| 6 | *Pseudognaptorina himalayana* **sp. nov.** | Qucho Mountain, Lhunze County, Xizang, China (TiLZ01) | 4824 | 30.Ⅶ.2019 | X.-M. Li *et al*. | PV197105 |
| 7 | *Pseudognaptorina himalayana* **sp. nov.** | Qucho Mountain, Lhunze County, Xizang, China (TiLZ02) | 4824 | 30.Ⅶ.2019 | X.-M. Li *et al*. | PV197106 |
| 8 | *Pseudognaptorina himalayana* **sp. nov.** | Yardoigar la Mountain, Cimai County, Xizang China (TiCM) |  | 8.VIII.2014 | G.-D. Ren *et al*. | PV197107 |
| 9 | *Pseudognaptorina migana* **sp. nov.** | Mila mountain pass, Gongbogyamda County, Xizang, China (TiGBJD) | 4750 | 18.Ⅶ.2023 | X.-M. Li *et al*. | PV197109 |
| 10 | *Pseudognaptorina oblonga* **sp. nov.** | Lhari County, Xizang, China (TiJL04) |  | 22.Ⅶ.2013 | X.-L. Bai *et al*. | PV197098 |
| 11 | *Pseudognaptorina oblonga* **sp. nov.** | Arza Township, Lhari County, Xizang, China (TiJL01) | 4300 | 9.VIII.2019 | G.-D. Ren *et al*. | PV197099 |
| 12 | *Pseudognaptorina oblonga* **sp. nov.** | Arza Township, Lhari County, Xizang, China(TiJL02) | 4300 | 9.VIII.2019 | G.-D. Ren *et al*. | PV197097 |
| 13 | *Pseudognaptorina oblonga* **sp. nov.** | Lhari County, Xizang, China (TiJL03) | 4762 | 17.Ⅶ.2023 | X.-M. Li *et al*. | PV197100 |
| 14 | *Pseudognaptorina rectangularis* **sp. nov.** | Hongyuan County, Sichuan, China (SCHY) | 3495 | 22.Ⅶ.2021 | X.-M. Li *et al*. | PV197113 |
| 15 | *Pseudognaptorina reni* **sp. nov.** | Gongri Township, Baqen County, Xizang, China (TiBQ) | 4556 | 4.Ⅶ.2022 | G.-D. Ren *et al*. | PV197110 |
| 16 | *Pseudognaptorina reni* **sp. nov.** | Vajra Pass, Zadowa County, Qinghai, China(QHZD01) | 4718 | 26.Ⅶ.2019 | G.-D. Ren *et al*. | PV197111 |
| 17 | *Pseudognaptorina reni* **sp. nov.** | Vajra Pass, Zadowa County, Qinghai, China (QHZD02) | 4718 | 26.Ⅶ.2019 | G.-D. Ren *et al*. | PV197112 |
| 18 | *Gnaptprina australis* | Shangri-la County, Yunnan, China（YNSL） |  | 26.Ⅵ.2019 |  | PV197096 |
| 19 | *Gnaptprina australis* | Baima snow Mountain, Deqin County, Yunnan, China (YNDQ) | 4328 | 23.Ⅴ.2023 | X.-L. Bai *et al*. | PV197095 |
| 20 | *Agnaptoria markana* | Riwoqe County, Xizang, China (TiLWQ) | 4564 | 28.Ⅶ. 2019 | G.-D. Ren *et al*. | PV197138 |
| 21 | *Agnaptoria markana* | Zogang County, Xizang, China (TiZG) | 3808 | 9. Ⅷ. 2015 | G.-D. Ren *et al*. | PV197136 |
| 22 | *Agnaptoria markana* | Marxog, Banbar County, Xizang, China (TiBB02) | 4400 | 31.Ⅶ. 2019 | G.-D. Ren *et al*. | PV197139 |
| 23 | *Agnaptoria markana* | Gadingla shan, Banbar County, Xizang, China (TiBB01) | 5140 | 12. Ⅶ. 2015 | G.-D. Ren *et al*. | PV197137 |
| 24 | *Agnaptoria markana* | Biru, Biru County, Xizang, China (TiBR) | 4496 | 1. Ⅷ. 2019 | G.-D. Ren *et al*. | PV197140 |
| 25 | *Agnaptoria ruida* | Haizi mountain, Batang County, Sichuan, China (SCBT02) | 4448 | 13. Ⅷ. 2016 | X.-M. Li *et al*. | PV197143 |
| 26 | *Agnaptoria ruida* | Haizi mountain, Batang County, Sichuan, China (SCBT03) | 4566 | 13. Ⅷ. 2016 | X.-M. Li *et al*. | PV197142 |
| 27 | *Agnaptoria ruida* | Shangdeda Township, Batang County, Sichuan, China (SCBT04) |  | 15.Ⅷ. 2014 | G.-D. Ren *et al*. | PV197144 |
| 28 | *Agnaptoria kozlovi* | Zhuodala shan, Garzê County, Sichuan, China (SCGZ) | 4800 | 5. Ⅷ. 2016 | X.-M. Li *et al*. | PV197141 |
| 29 | *Agnaptoria grummi* | Sitongda Township, Garzê County, Sichuan, China (SCGZ) | 4517 | 4. Ⅷ. 2016 | X.-M. Li *et al*. | PV197145 |
| 30 | *Agnaptoria anthracina anthracina* | Que er shan, Dêgê County, Sichuan, China (SCDG) | 4707 | 18. Ⅵ. 2016 | X.-B. Guo | PV197146 |
| 31 | *Agnaptoria minuta* | Makehe Township, Banma County, Qinghai, China (QHBM) |  | 26. Ⅵ. 2013 | F.-M. Shi | PV197158 |
| 32 | *Agnaptoria rubripes* | Shiqu County, Sichuan, China (SCSQ) |  | 2. VII. 2022 | G.-D. Ren *et al*. | PV197148 |
| 33 | *Agnaptoria* sp.1 | Nata Township, Baiyü County, Sichuan, China (SCBY) | 4100 | 5. Ⅷ. 2016 | X.-M. Li *et al*. | PV197149 |
| 34 | *Agnaptoria* sp.2 | Chowa Township, Dêgê County, Sichuan, China (SCDG) | 4007 | 24. Ⅷ. 2018 | X. -L. Bai *et al*. | PV197150 |
| 35 | *Agnaptoria miroshnikovi* | Kazila mountain, Litang County, Sichuan, China (SCLT) | 4416 | 15. Ⅷ. 2016 | X.-M. Li *et al*. | PV197151 |
| 36 | *Agnaptoria miroshnikovi* | Yajiang County, Sichuan, China (SCYJ) |  | 9. Ⅶ. 2012 | H. Huang | PV197152 |
| 37 | *Agnaptoria* sp.3 | Sangdui Township, Daocheng County, Sichuan, China (SCDC) | 4584 | 26. VII. 2020 | M.-M. Ma | PV197153 |
| 38 | *Agnaptoria gansuensis* | Huangcheng Township, Menyuan County, Qinghai, China (QHMY01) |  | 13. Ⅷ. 2019 | Z.-J. Zhou | PV197155 |
| 39 | *Agnaptoria gansuensis* | Laohu ditch, Menyuan County, Qinghai, China (QHMY02) | 3550 | 27. VII. 2021 | X. -L. Bai *et al*. | PV197157 |
| 40 | *Agnaptoria gansuensis* | Qingyang ditch, Qilian County, Qinghai, China (QHQL) | 3148 | 1. VIII. 2021 | X.-L. Bai *et al*. | PV197156 |
| 41 | *Agnaptoria belousovi* | Makehe Township, Banma County, Qinghai, China (QHBM) |  | 27. Ⅵ. 2013 | F.-M. Shi | PV197147 |
| 42 | *Agnaptoria lauta* | Wanglang National Nature Reserve, Pingwu County, Sichuan, China（SCPW） |  | 29. VII. 2021 | Y.-Y. Qin | PV197159 |
| 43 | *Agnaptoria abdita* | Maqu County, Gansu, China (GSMQ) |  | 2022-7-1 | G.-D. Ren *et al*. | PV197160 |
| 44 | *Agnaptoria danbaensis* | Zheduo moutain, Kangding County, Sichuan, China (SCKD) | 4300 | 2022-06-27 | J.-S. Hao | PV197154 |
| 45 | *Agnaptoria nigriceps* | Cawarong Township, Zayü County, Xizang, China (TiZY01) | 4608 | 19. VII. 2021 | G.-D. Ren *et al*. | PV197162 |
| 46 | *Agnaptoria nigriceps* | Zhuwagen Township, Zayü County, Xizang, China (TiZY02) | 4568 | 29. Ⅶ. 2017 | X.-L. Bai *et al*. | PV197164 |
| 47 | *Agnaptoria nigriceps* | Zhuwagen Township, Zayü County, Xizang, China (TiZY03) | 3753 | 4. V. 2023 | X.-L. Bai *et al*. | PV197163 |
| 48 | *Agnaptoria nyingcha* | Zhamog Township, Bome County, Xizang, China (TiBM01) | 3360 | 1. Ⅷ. 2017 | X.-L. Bai *et al*. | PV197161 |
| 49 | *Agnaptoria nyingcha* | Zhamog, Bome County, Xizang, China (TiBM02) | 3142 | 25. VII. 2021 | G.-D. Ren *et al*. | PV197165 |
| 50 | *Agnaptoria nyingcha* | Hongla mountain pass, Markom county, Xizang, China (TiMK) | 4155 | 2023-5-23 | X.-L. Bai *et al*. | PV197166 |
| 51 | *Asidoblaps zamotailovi* | Shangri-La, Yunan, China (YNSL01) |  | Ⅵ.2023 |  | PV197114 |
| 52 | *Asidoblaps zamotailovi* | Shangri-La, Yunan, China (YNSL02) |  | Ⅵ.2023 |  | PV197115 |
| 53 | *Asidoblaps zamotailovi* | Shangri-La, Yunan, China (YNSL03) |  | Ⅵ.2023 |  | PV197116 |
| 54 | *Asidoblaps sinensis* | Kangding County, Sichuan, China (SCKD) | 3170 | 30.Ⅸ.2022 | L. Wang *et al*. | PV197130 |
| 55 | *Asidoblaps bianera* | Dawu County, Sichuan, China (SCDW) |  | 3.Ⅷ.2022 | Z. Wei *et al*. | PV197129 |
| 56 | *Asidoblaps rilonga* | Songpan County, Sichuan, China (SCSP) | 3501 | 30.Ⅸ.2022 | Y. Qing *et al*. | PV197128 |
| 57 | *Asidoblaps confinis* | Xiaojin County, Sichuan, China (SCXJ) | 2954 | 30.Ⅶ.2009 | Y. Niu *et al*. | PV197133 |
| 58 | *Asidoblaps confinis* | Danba County, Sichuan, China (SCDB) |  | 2.Ⅷ.2000 | Y. Niu *et al*. | PV197134 |
| 59 | *Asidoblaps attigua* | Zayü County, Xizang, China (TiZY02) | 3256 | 18.VII.2021 | G. Ren *et al*. | PV197118 |
| 60 | *Asidoblaps attigua* | Zayü County, Xizang, China (TiZY01) | 3304 | 17.VII.2021 | G. Ren *et al*. | PV197117 |
| 61 | *Asidoblaps konstantinovi* | Weixi Lisuzu County, Yunan, China (YNWX) | 3300 | 15.VII.2021 | Z. Wei | PV197119 |
| 62 | *Asidoblaps justa* | Hongyuan County, Sichuan, China (SCHY02) | 3451 | 20.VII.2021 | Z. Wei | PV197125 |
| 63 | *Asidoblaps justa* | Shuajingsi, Sichuan, China (SCSJS) |  | 27.VII.2022 | Z. Wei | PV197123 |
| 64 | *Asidoblaps justa* | Hongyuan County, Sichuan, China (SCHY01) |  | 24.VII.2011 | G. Ren *et al*. | PV197122 |
| 65 | *Asidoblaps justa* | Hongyuan County, Sichuan, China (SCHY02) | 3451 | 20.VII.2021 | X. Li *et al*. | PV197124 |
| 66 | *Asidoblaps gorgneri* | Hongyuan County, Sichuan, China (SCHY03) | 2705 | 9.VIII.2021 | Y. Qin *et al*. | PV197131 |
| 67 | *Asidoblaps gorgneri* | Pingwu County, Sichuan, China (SCPW) | 2508 | 31.VII.2021 | Y. Qin *et al*. | PV197132 |
| 68 | *Asidoblaps modica* | Songpan County, Sichuan, China (SCSP) |  | 21.VII.2022 | Z. Wei *et al*. | PV197135 |
| 69 | *Asidoblaps subnotata* | Li County, Sichuan, China (SClX01) | 3533 | 19.VII.2021 | X. Li *et al*. | PV197126 |
| 70 | *Asidoblaps subnotata* | Li County, Sichuan, China (SCLX02) | 3533 | 19.VII.2021 | X. Li *et al*. | PV197127 |
| 71 | *Asidoblaps pedinoides* | Daocheng County, Sichuan, China (SCDC) | 4466 | 6.VIII.2017 | X. Bai *et al*. | PV197120 |
| 72 | *Asidoblaps yaanensis* | Baoxing County, Sichuan, China (SCBX) |  | 29.Ⅵ.2012 | H. Huang *et al*. | PV197121 |
